# Healthcare- and Community-Associated Methicillin-Resistant *Staphylococcus aureus* (MRSA) and Fatal Pneumonia with Pediatric Deaths in Krasnoyarsk, Siberian Russia: Unique MRSA's Multiple Virulence Factors, Genome, and Stepwise Evolution

**DOI:** 10.1371/journal.pone.0128017

**Published:** 2015-06-05

**Authors:** Olga E. Khokhlova, Wei-Chun Hung, Tsai-Wen Wan, Yasuhisa Iwao, Tomomi Takano, Wataru Higuchi, Svetlana V. Yachenko, Olga V. Teplyakova, Vera V. Kamshilova, Yuri V. Kotlovsky, Akihito Nishiyama, Ivan V. Reva, Sergey V. Sidorenko, Olga V. Peryanova, Galina V. Reva, Lee-Jene Teng, Alla B. Salmina, Tatsuo Yamamoto

**Affiliations:** 1 Russia-Japan Center of Microbiology, Metagenomics and Infectious Diseases, Krasnoyarsk State Medical University named after Professor V.F. Vojno-Yasenetsky, Krasnoyarsk, Russia; 2 Department of Microbiology, Krasnoyarsk State Medical University named after Professor V.F. Vojno-Yasenetsky, Krasnoyarsk, Russia; 3 Division of Bacteriology, Department of Infectious Disease Control and International Medicine, Niigata University Graduate School of Medical and Dental Sciences, Niigata, Japan; 4 Department of Epidemiology, Genomics, and Evolution, International Medical Education and Research Center, Niigata, Japan; 5 Department of Clinical Laboratory Sciences and Medical Biotechnology, National Taiwan University College of Medicine, Taipei, Taiwan; 6 Department of Microbiology and Immunology, Kaohsiung Medical University, Kaohsiung, Taiwan; 7 Krasnoyarsk Regional Center for Prevention and Control of AIDS and Infectious Diseases, Krasnoyarsk, Russia; 8 City Clinical Hospital No. 7, Krasnoyarsk, Russia; 9 Department of General Surgery, Krasnoyarsk State Medical University named after Professor V.F. Vojno-Yasenetsky, Krasnoyarsk, Russia; 10 Municipal Hospital Emergency Medical Care named after N.S. Karpovich, Krasnoyarsk, Russia; 11 Central Research Laboratory of Krasnoyarsk State Medical University named after Professor V.F. Vojno-Yasenetsky, Krasnoyarsk, Russia; 12 Far Eastern Federal University School of Biomedicine, Vladivostok, Russia; 13 Research Institute of Children’s Infection, St. Petersburg, Russia; 14 Research Institute of Molecular Medicine and Pathobiochemistry, Krasnoyarsk State Medical University named after Professor V.F. Vojno-Yasenetsky, Krasnoyarsk, Russia; University Hospital Münster, GERMANY

## Abstract

Methicillin-resistant *Staphylococcus aureus* (MRSA) is a common multidrug-resistant (MDR) pathogen. We herein discussed MRSA and its infections in Krasnoyarsk, Siberian Russia between 2007 and 2011. The incidence of MRSA in 3,662 subjects was 22.0% and 2.9% for healthcare- and community-associated MRSA (HA- and CA-MRSA), respectively. The 15-day mortality rates for MRSA hospital- and community-acquired pneumonia (HAP and CAP) were 6.5% and 50%, respectively. MRSA CAP cases included pediatric deaths; of the MRSA pneumonia episodes available, ≥27.3% were associated with bacteremia. Most cases of HA-MRSA examined exhibited ST239/*spa*3(t037)/SCC*mec*III.1.1.2 (designated as ST239_Kras_), while all CA-MRSA cases examined were ST8/*spa*1(t008)/SCC*mec*IV.3.1.1(IVc) (designated as ST8_Kras_). ST239_Kras_ and ST8_Kras_ strongly expressed cytolytic peptide (phenol-soluble modulin α, PSMα; and δ-hemolysin, Hld) genes, similar to CA-MRSA. ST239_Kras_ pneumonia may have been attributed to a unique set of multiple virulence factors (MVFs): toxic shock syndrome toxin-1 (TSST-1), elevated PSMα/Hld expression, α-hemolysin, the staphylococcal enterotoxin SEK/SEQ, the immune evasion factor SCIN/SAK, and collagen adhesin. Regarding ST8_Kras_, SEA was included in MVFs, some of which were common to ST239_Kras_. The ST239_Kras_ (strain OC3) genome contained: a completely unique phage, φSa7-like (W), with no *att* repetition; *S*. *aureus* pathogenicity island SaPI2R, the first TSST-1 gene-positive (*tst* ^+^) SaPI in the ST239 lineage; and a super copy of IS*256* (≥22 copies/genome). ST239_Kras_ carried the Brazilian SCC*mec*III.1.1.2 and United Kingdom-type *tst*. ST239_Kras_ and ST8_Kras_ were MDR, with the same levofloxacin resistance mutations; small, but transmissible chloramphenicol resistance plasmids spread widely enough to not be ignored. These results suggest that novel MDR and MVF^+^ HA- and CA-MRSA (ST239_Kras_ and ST8_Kras_) emerged in Siberian Russia (Krasnoyarsk) associated with fatal pneumonia, and also with ST239_Kras_, a new (Siberian Russian) clade of the ST239 lineage, which was created through stepwise evolution during its potential transmission route of Brazil-Europe-Russia/Krasnoyarsk, thereby selective advantages from unique MVFs and the MDR.

## Introduction

Methicillin-resistant *Staphylococcus aureus* (MRSA) has been a major multidrug-resistant (MDR) pathogen since the early 1960s [1], with recent threats, such as intensive care unit (ICU)-associated bacteremia in London [2], serious invasive infections in the United States (US) [3], and global antimicrobial resistance in common infections, being alerted by the World Health Organization (WHO) [4].

Traditional MRSA is now classified as healthcare-associated MRSA (HA-MRSA) [3], and another class of MRSA, which emerged in community settings between 1997 and 1999, as community-associated MRSA (CA-MRSA) [3,5,6]. HA- and CA-MRSA both carry staphylococcal cassette chromosome *mec* (SCC*mec*) [7,8], and each have several genetic backgrounds [9–14], which are generally identified based on multilocus sequence types (ST types), protein A gene (*spa*) types, and SCC*mec* types [6,8,12,14].

The most disseminated HA-MRSA worldwide includes the ST239 lineage, such as ST239/SCC*mec*IIIA [15] and ST239/SCC*mec*III [2,16–20], as well as the ST5 lineage, such as ST5/SCC*mec*II carrying the toxic shock syndrome toxin-1 (TSST-1) gene (*tst*) [21,22]. The most characterized CA-MRSA includes the ST8 lineage, such as ST8/SCC*mec*IVa (USA300) [12,23], and also the lineages of ST30/SCC*mec*IV [6,24–26], ST59/SCC*mec*V [27–29], and ST80/SCC*mec*IV [6,24,30]. Although CA-MRSA, unlike HA-MRSA, is generally less MDR [31], CA-MRSA also has the capacity to become MDR (representative, USA300) [32,33]. Some CA-MRSA are MDR from their emergence (representative, the Taiwan clone) [29].

HA-MRSA infections most frequently occur among inpatients [34,35], while CA-MRSA infections occur in healthy individuals. CA-MRSA mainly causes skin and soft tissue infections (SSTIs), but also occasionally invasive infections [6,13,34,36]. The median ages of HA-MRSA and CA-MRSA patients are 68 and 23 years, respectively [34]. CA-MRSA often produces Panton-Valentine leukocidin (PVL) [6,12,13,37–39], and exhibits elevated expression of cytolytic peptides (phenol-soluble modulins, PSMs; or δ-hemolysin, Hld) [12,40].

MRSA evolution includes horizontal gene transfer, mediated by mobile genetic elements, plasmids, and phages, and also through mutations [8,37,41–44]. The mosaicism of the genome has also been reported; ST239 MRSA is a bacterial hybrid between clonal complex (CC) 30 (founder, ST30) and CC8 (founder, ST8) [19,45]. MRSA occasionally spreads by intercontinental transmission [9,20,46], and replacement often occurs [18,47,48]. The evolution of MRSA is still dynamic, and, therefore, may attack humans posing a new threat.

In Russia, dominant MRSA are ST239/SCC*mec*III and PVL-negative (PVL^-^) ST8/SCC*mec*IV [49]. Although we previously reported PVL-positive (PVL^+^) ST30 CA-MRSA [50], *tst* ^+^ ST239 HA-MRSA [51], and a whole genome structure [52], information on MRSA in Russia is still limited at the molecular level, especially in Siberian Russia, which is located between the European and Far Eastern regions. In the present study, we focused on episodes (and mortality rates) of MRSA hospital-acquired pneumonia (HAP) and community-acquired pneumonia (CAP) with pediatric deaths in Krasnoyarsk, Siberian Russia, as has been reported with initial fatal pediatric MRSA CAP episodes in the US North areas [5]. We discussed possible MRSA multiple virulence factors (MVFs), implicated in fatal cases of MRSA HAP and CAP. We then demonstrated their unique genomic structures and evolution of representative fatal-pneumonia-associated MRSA.

## Materials and Methods

### Ethics statement

The Ethics Review Boards of Krasnoyarsk State Medical University (Ethics Review Board No28/2010), Krasnoyarsk, Russia; Far Eastern Federal University School of Biomedicine, Vladivostok, Russia; National Taiwan University College of Medicine, Taipei, Taiwan; Niigata University School of Medicine, Niigata, Japan; and International Medical Education and Research Center, Niigata, Japan, specifically approved this study. Written informed consent was obtained from patients, if necessary.

### Patients and bacterial strains

A total of 3,662 subjects were examined in Krasnoyarsk between 2007 and 2011. *S*. *aureus* specimens including MRSA were isolated in four hospitals in Krasnoyarsk, and all bacterial strains were isolated from different individuals. The data obtained are summarized in Table 1. The follow-up period used to determine the mortality for pneumonia was 15 days in this study; and 15-day mortality rates were compared between MRSA HAP and MRSA CAP cases. HA-MRSA was defined as MRSA isolated from inpatients 48 h after hospitalization while CA-MRSA was defined as MRSA isolated from outpatients who had no history of hospitalization within at least the past year and presented with no other established risk factors for MRSA infections [3].

**Table 1:** Clinical and bacteriological information on MRSA isolated in Krasnoyarsk between 2007 and 2011.

Study group	Diseases	Subjects		Bacterial isolation				Fatal cases		
		Total number	Age (Y)	Isolation year	*S*. *aureus*(MRSA)	MRSA/total subjects	MRSA/*S*. *aureus*	/subjects	/*S*. *aureus*	/MRSA
Inpatients	Pneumonia	710	0–81	2007–2011^b^	221 (62)	8.7%	28.1%	0.6%	0%	6.5%^c^
						(62/710)	(62/221)	(4/710)	(0/159)	(4/62)
	SSTIs^a^	874	20–84	2010–2011	210 (31)	3.5%	14.8%	0.1%	0%	3.2%
						(31/874)	(31/210)	(1/874)	(0/179)	(1/31)
	Osteomyelitis	208	21–80	2010–2011	77 (19)	9.1%	24.7%	0%	0%	0%
						(19/208)	(19/77)	(0/208)	(0/58)	(0/19)
Outpatients	Pneumonia	310	0–67	2007–2008	93 (8)	2.6%	8.6%	1.3%	0%	50%^c^
						(8/310)	(8/93)	(4/310)	(0/85)	(4/8)
	SSTIs^a^	126	27–84	2010–2011	41 (2)	1.6%	4.9%	0%	0%	0%
						(2/126)	(2/41)	(0/126)	(0/39)	(0/2)
	Colitis	401	0–9	2011	357 (4)	1.0%	1.1%	0%	0%	0%
						(4/401)	(4/357)	(0/401)	(0/353)	(0/4)
Carriers	Students in the	287	18–20	2010–2011	77 (1)	0.3%	1.3%	0%	0%	0%
	community					(1/287)	(1/77)	(0/287)	(0/76)	(0/1)
	Athletes in the	108	11–28	2011	42 (0)	0%	0%	0%	0%	0%
	community					(0/108)	(0/42)	(0/108)	(0/42)	(0/0)
	Hospital	638	22–52	2008–2011	183 (2)	0.3%	1.1%	0%	0%	0%
	workers					(2/638)	(2/183)	(0/638)	(0/181)	(0/2)

^a^SSTIs, skin and soft tissue infections; in this study, SSTIs include dactylitis, paronychia, hidradenitis, wound infection, skin abscess, furuncle, carbuncle, bursitis, cellulitis, erysipelas-like necrotic cellulitis, and necrotizing fasciitis.

^b^Number of MRSA isolates in 2007–2009 was 42, and that in 2010–2011 was 20 (total 62).

^c^ *P*<0.01

Russian MRSA strains also included ten strains from inpatients (age, 1–41 years) with burn wound infections and respiratory tract infections in Vladivostok in 2012 and 2013; and nine strains from patients with burn and wound infections, osteomyelitis, respiratory tract infections, and blood stream infections in Moscow and Saint-Petersburg (European Russia) and in Kurgan (Ural Federal Region, Russia) in 2011 and 2012.

The following were used as reference or control strains. Strain RS08 (PVL^+^ ST30/*spa*19-t019/SCC*mec*IVc) was isolated from a female badminton player in her twenties with furunculosis in Vladivostok in 2006 [50]. Of the ST239/*spa*351(t030)/ SCC*mec*III.1.1.4 strains, 16K was isolated from a 20-year-old male outpatient with urethritis in Vladivostok between 2006 and 2008 [52]. Another 11 strains were collected from Vladivostok [52]. Of the ST239/*spa*3(t037)/SCC*mec*III.1.1.1 MRSA (*tst* ^-^) strains, nine (including strain 6K) were from Vladivostok [52] and four (PM3, PM14, PM27, and PM38) were from Taiwan [28]. Of the PVL^+^ ST8/SCC*mec*IVa CA-MRSA (USA300) strains, USA300-0114 was a type strain kindly provided by L. K. McDougal and L. L. McDonald and NN36 [53] and NN47 [54] were Japanese isolates. Of the *tst* ^+^ ST5/SCC*mec*II HA-MRSA (NY/J clone) strains, N315 and Mu50 were reference strains that were kindly provided by K. Hiramatsu, I6 was a Japanese isolate [55], and PM29 was a Taiwanese isolate [28]. The reference strains HU25 (ST239/SCC*mec*III.1.1.2-IIIA, Brazilian clone) and ANS46 (ST239/SCC*mec*III.1.1.1) were kindly provided by H. de Lencastre.

### Genotyping and virulence gene analysis

ST typing, CC assignment, *spa* typing, *agr* typing, and SCC*mec* typing were performed as described previously [8,56]. Coagulase (Coa) typing was conducted using a staphylococcal coagulase antiserum kit (Denka Seiken, Tokyo, Japan). Virulence genes were analyzed by PCR [56]; the target genes in PCR included 49 genes: 3 leukocidin genes (*luk*_*PV*_ *SF*, *lukE-lukD*, and *lukM*), 5 hemolysin genes (*hla*, *hlb*, *hlg*, *hlg-v*, and *hld*), a peptide cytolysin (*psmα*), 19 staphylococcal enterotoxin (SE) genes (*tst*, *sea*, *seb*, *sec*, *sed*, *see*, *seg*, *seh*, *sei*, *sej*, *sek*, *sel*, *sem*, *sen*, *seo*, *sep*, *seq*, *ser*, and *set*), 1 putative SE gene (*seu*), 3 exfoliative toxin genes (*eta*, *etb*, and *etd*), a staphylococcal superantigen-like gene cluster (*ssl*), the epidermal cell differentiation inhibitor gene (*edin*), 14 adhesin genes (*icaA*, *icaD*, *eno*, *fib*, *fnbA*, *fnbB*, *ebpS*, *clfA*, *clfB*, *sdrC*, *sdrD*, *sdrE*, *cna*, and *bbp*), and the arginine catabolic mobile element (ACME)-*arcA* gene.

### TSST-1 and SEA assays

The amounts of TSST-1 and SEA in the supernatants of bacterial cultures at 2.0 X l0^9^ CFU/ml were examined using a TST- RPLA kit (Denka Seiken) and SET-RPLA kit (Denka Seiken), respectively, according to the instructions of the manufacturer.

### Pulsed-field gel electrophoresis (PFGE) analysis

Bacterial DNA for PFGE was digested with *Sma*I and electrophoresed in 1.2% agarose with marker DNA (Lambda ladder; Bio-Rad Laboratories, Inc., Hercules, CA, USA), as described previously [52].

### Plasmid analysis

The plasmid DNA of MRSA was prepared using a Plasmid Midi Kit (QIAGEN Sciences, Tokyo) or according to the method by Kado and Liu [57] with a modification to the lysostaphin treatment. Plasmid DNA was analyzed by agarose (0.6–1.0%) gel electrophoresis. The Tn*554* circular intermediate was detected by PCR (PCR product size, 772 bp), as previously described [52].

### Conjugative transfer

Donor strains were mated with *S*. *aureus* RN2677, a recipient strain, which is resistant to rifampicin (Rif^r^) and novobiocin and carries no plasmids, on tryptic soy agar (Difco, Sparks, MD, USA), with or without membrane filters [52].

### Susceptibility testing

Susceptibility testing of bacterial strains was performed using the agar dilution method with Mueller-Hinton agar [58]. Inducible clindamycin resistance (Cli^r^) was tested, as above, by using agar plates containing erythromycin (Em) at 0.1 to 1 μg/ml.

### Drug resistance gene analysis

The genes for drug resistance, antiseptic resistance, and heavy metal resistance were analyzed by PCR [28,29]. The genes (resistance phenotypes) analyzed were: *mecA* (resistance to methicillin, oxacillin, and cephems), *blaZ* (resistance to ampicillin), *ermA* and *ermC* (Em^r^/Cli^r^), *cat* (resistance to chloramphenicol, Cp^r^), *aacA-aphD* (resistance to gentamicin, Gm^r^, and kanamycin), *aadD* (resistance to neomycin), *tet* (resistance to tetracycline), *spc* (spectinomycin, Spc^r^), *ble* (resistance to bleomycin), *qacA* (resistance to acriflavin/quaternary ammonium, such as benzalkonium chloride and benzethonium chloride/chlorhexidine gluconate/ethidium bromide), *cad* (resistance to cadmium), and *mer* (resistance to mercury).

### Genome analysis

The ST239 MRSA OC3 genome was analyzed by pyrosequencing using a genome sequencer FLX system with the assembler software GS *De Novo* Assembler version 2.6 (Roche Diagnostics, Branford, CT, USA). The GenBank accession numbers for the OC3 genome (144 contigs with ≥20 bp in size) are BBKC01000001-BBKC01000144. The OC3 contigs were mapped on the 3,043,210-bp complete genome (GenBank accession number FN433596) of TW20 (the most characterized ST239) using MUMmer software (http://mummer.sourceforge.net/). The gene or open reading frame (*orf*) was searched for using the software in silico MolecularCloning (version 4.2) (In Silico Biology, Yokohama, Japan).

### Entire sequencing of mobile genetic elements, phages, and plasmids

The gaps between contigs were filled by PCR and sequencing. We also assembled contigs using an LA PCR *in vitro* cloning kit (Takara Bio, Shiga, Japan) according to the manufacturer’s instructions. In brief, after digestion with suitable restriction enzymes and ligation with the corresponding cassette adapters, amplification was performed with cassette primers and target-specific primers.

### Phylogenetic and homology analyses

Multiple alignments were performed up to 1,000 times using default settings with ClustalW software (version 2.1) and a phylogenetic tree analysis was performed using TreeViewX software (version 0.5.0) (http://taxonomy.zoology.gla.ac.uk/rod/treeview.html). A homology analysis was performed using the software BLAST (http://blast.ddbj.nig.ac.jp/top-e.html) and FASTA (http://fasta.ddbj.nig.ac.jp/top-j.html).

### mRNA expression assay

The mRNA expression levels of the cytolytic peptide (PSMα and Hld) genes (*psmα* and *hld*) and 16S rRNA genes were examined by an RT-PCR assay [56,59]. *psmα* and *hld* expression levels were normalized to 16S rRNA expression levels. The mRNA expression levels of the transcriptional regulator genes (*sarA*, *sarR*, *mgrA*, *saeR*, *saeS*, *sarX*, *rot*, *and srrAB*) were also examined.

### Statistical analysis

Data were evaluated by Fisher’s exact test for MRSA incidence and by an analysis of variance with repeated measurements for the mRNA expression assay. The level of significance was defined as a *P* value of <0.05. Regarding 15-day mortality rate estimates, 95% confidence intervals (95% CIs) were included.

## Results

### MRSA infection in Krasnoyarsk, Siberian Russia

A total of 3,662 subjects were examined for *S*. *aureus* and MRSA infections, and the data obtained are summarized in Table 1. Regarding nosocomial infections (inpatients), MRSA infections accounted for 8.7% (62/710) of cases of pneumonia, 3.5% (31/874) of SSTIs, and 9.1% (19/208) of osteomyelitis; the overall incidence of MRSA (among *S*. *aureus* isolates) was 22.0% (112/508). Among the cases of MRSA HAP, 6.5% (4/62) were fatal (Tables 1 and 2); the ages of these four patients were 39, 46, 48, and 71 years old (average, 51 years old), and these deaths occurred in 2007 (three cases) and 2011 (one case). One fatal case of nosocomial SSTIs (3.2%, 1/31) was noted and may have been due to sepsis.

**Table 2:** List of MRSA strains characterized at molecular levels in the present study^a^.

Isolation year	Name ofMRSA	ST type (epidemiologicalclassification of MRSA)	Patients			
			Disease	Outcome	Age	Sex
2007	OC3	ST239 (HA)	Pneumonia, sepsis (bacteremia)	Death	46Y	M
	OC76	ST239 (HA)	Pneumonia	Death	71Y	F
	OC8	ST8 (CA)	Pneumonia	Death	1Y	M
	OC11	ST8 (HA)	Pneumonia, sepsis (bacteremia)	Death	39Y	M
2008	OC22	ST8 (CA)	Pneumonia	Death	41Y	M
	OC23	ST8 (CA)	Pneumonia	Death	40Y	M
	OC59	ST8 (CA)	Pneumonia	Death	4M	F
	OC52	ST8 (HA/CA)	-^b^	-^b^	34Y	F
2009	OC180	ST239 (HA)	Pneumonia	Recovery	34Y	M
2010	OC22B	ST239 (HA)	Osteomyelitis	Recovery	22Y	M
	OC70	ST239 (HA)	Osteomyelitis	Recovery	40Y	M
	OC66	ST239 (HA)	Erysipelas-like necrotic cellulitis	Recovery	59Y	F
	OC159	ST239 (HA)	Erysipelas-like necrotic cellulitis	Recovery	57Y	F
	OC114	ST239 (HA)	Wound infection, cellulitis	Recovery	58Y	M
	OC145	ST239 (HA)	Wound infection	Recovery	50Y	M
	OC1	ST8 (CA)	Skin abscesses	Recovery	50Y	F
	OC217	ST8 (CA)	-^c^	-^c^	19Y	F
	OC50	ST12 (HA)	Surgical site infection, (sepsis)	Death	84Y	M
2011	OC8C	ST239 (HA)	Pneumonia	Death	48Y	M
	OC14	ST239 (HA)	Pneumonia	-^d^	-^d^	F
	OC35	ST239 (HA)	Pneumonia, sepsis (bacteremia)	Recovery	27Y	M
	OC98	ST239 (HA)	Osteomyelitis	-^d^	31Y	M
	OC99	ST239 (HA)	Osteomyelitis	Recovery	30Y	M
	OC111	ST239 (HA)	Osteomyelitis	Recovery	76Y	F
	OC1A	ST239 (HA)	Burn infection	Recovery	37Y	M
	OC2	ST239 (HA)	Burn infection	Recovery	53Y	M
	OC5	ST239 (HA)	Wound infection	Recovery	31Y	M
	OC44	ST239 (HA)	Peritonitis	Recovery	30Y	M
	OC14C	ST239 (HA/CA)	-^b^	-^b^	43Y	F
	OC160	ST8 (CA)	Wound infection, cellulitis	Recovery	53Y	M
	OC1C	ST8 (CA)	Colitis	Recovery	3Y	M

^a^Y, years; M, male; F, female; HA, healthcare-associated MRSA; CA, community- associated MRSA.

^b^Healthy carrier (hospital worker)

^c^Healthy carrier (student)

^d^Information not available.

Among the cases of community-acquired infections (outpatients), MRSA infections accounted for 2.6% (8/310) of cases of pneumonia, 1.6% (2/126) of SSTIs, and 1.0% (4/401) of colitis; the overall incidence of MRSA (among *S*. *aureus* isolates) was 2.9% (14/491). The fatal case of MRSA CAP was 50% (4/8) (Tables 1 and 2); the ages of these four patients were 4 months old and 1, 40, and 41 years old (average, 20.5 years old), and these deaths occurred in 2007 (one case) and 2008 (three cases).

The incidence of MRSA (among *S*. *aureus* isolates) was significantly higher in hospitals (22%) than in the community (2.9%) (*P* <0.01). However, the 15-day mortality rates for MRSA CAP and MRSA HAP were 50% (95% CI, 17.5%-82.6%) and 6.5% (95% CI, 2.1%-16.5%), respectively (*P* <0.01).

Regarding healthy carriers (Table 1), MRSA^+^ cases were observed in 0.3% (2/638) of hospital workers in routine surveillance in hospitals, and in 0.3% (1/395) of students and athletes in occasional examinations in the community; the incidence of MRSA (among *S*. *aureus* isolates) was 1.1% (2/183) and 0.8% (1/119), respectively (*P* >0.05).

Among MRSA in Table 1, thirty-one isolates were subjected to molecular characterization (Table 2). When pneumonia was targeted between 2007 and 2009, eight MRSA were characterized, all of which were from fatal or severe cases only; MRSA was not available from other non-fatal pneumonia cases. When pneumonia, osteomyelitis, SSTIs, and colitis were mainly targeted in 2010 and 2011, MRSA from fatal cases were initially selected and characterized: two from MRSA HAP and nosocomial SSTI/sepsis. Eighteen isolates were randomly selected from non-fatal cases: two out of 19 pneumonia isolates, five out of 19 osteomyelitis isolates, nine out of 32 MRSA SSTI isolates, one out of four colitis isolates, and one isolate from peritonitis. All three MRSA isolates from healthy carriers were included in the molecular analysis.

In Table 2, at least three out of the 11 MRSA pneumonia episodes (pneumonia/sepsis) were associated with bacteremia with the incidence being ≥27.3%; at least two out of seven HAP episodes (>2/7) and one out of four CAP episodes (>1/4), or two out of six ST239 MRSA cases (>2/6) and one out of five ST8 MRSA cases (>1/5) were associated with bacteremia. Although two ST239 and ST8 HAP-related bacteremia cases in 2007 were fatal, one ST239 HAP-related bacteremia case in 2011 was not.

### Molecular characteristics of MRSA from Krasnoyarsk

**Fig 1:**
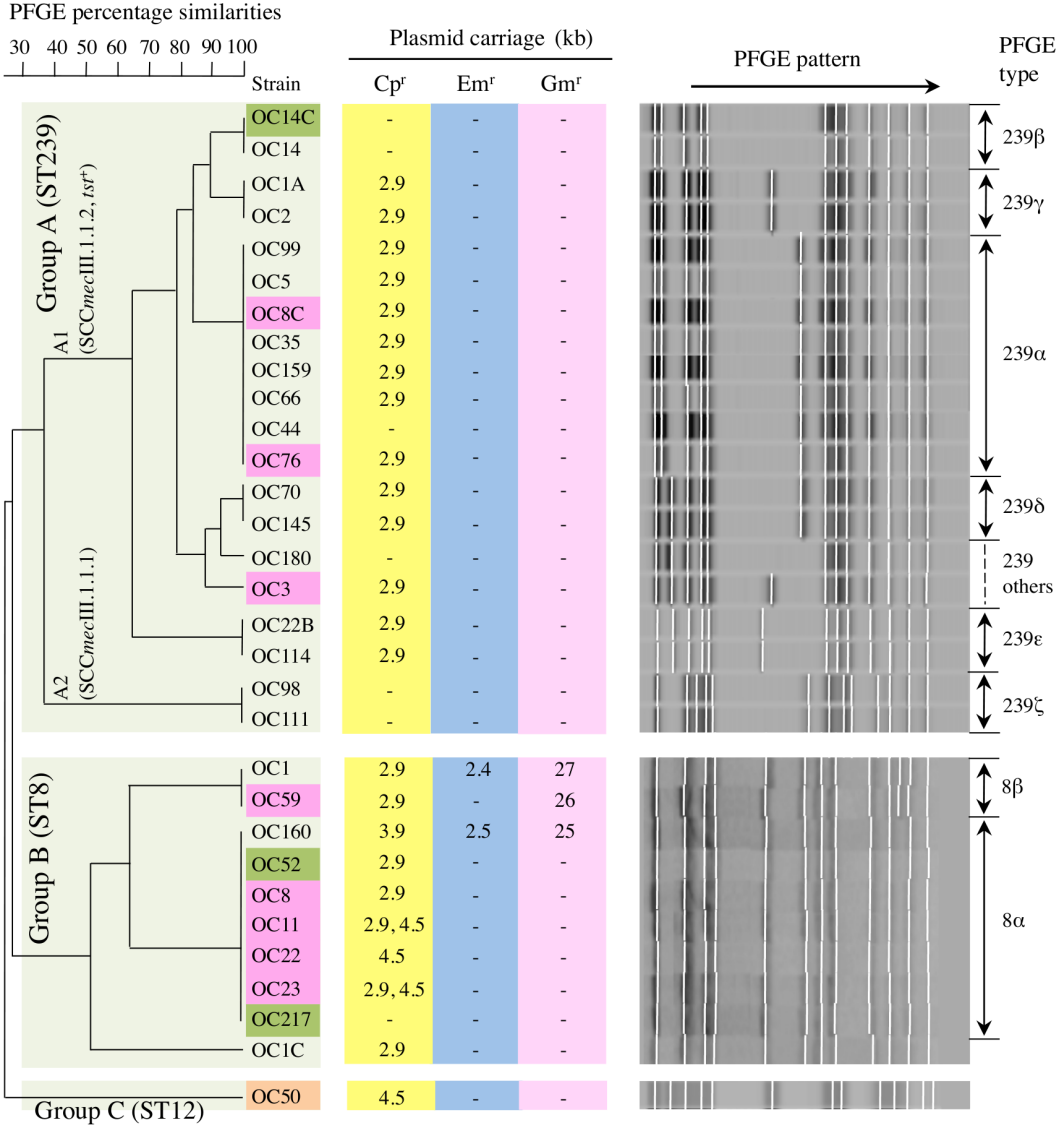
Pulsed-field gel electrophoresis (PFGE) analysis (right and left) and plasmid carriage patterns (center) of MRSA strains isolated in Krasnoyarsk. The MRSA strains shown are those described in Table 2. Group A (A1 and A2) and group B are described in Table 3. The color of the strain name indicates fatal pneumonia (red), possible sepsis (brown), and carrier cases (green). Of the cases of fatal pneumonia, OC3, OC8C, OC11, OC76 were from hospital-acquired pneumonia (HAP), while OC8, OC22, OC23, and OC59 were from community-acquired pneumonia (CAP). Of the carrier cases, OC14C and OC52 were from hospital workers and OC217 was from a student.

The molecular data of the 31 MRSA isolates are summarized in Table 3 and Fig 1. MRSA strains were classified into three groups, A to C (Table 3).

**Table 3:** Molecular characterization of MRSA strains isolated in Krasnoyarsk^a^.

Type, gene, or resistance	Group A		Group B	Group C
	A1 (n = 18)	A2 (n = 2)	(n = 10)	(n = 1)
Types				
CC	8	8	8	12
ST	239	239	8	12
*spa*	3 (t037)	3 (t037)	1 (t008)	new (t156)
*agr*	1	1	1	1
SCC*mec*	III.1.1.2	III.1.1.1	IV.3.1.1 (IVc)	UT
Coagulase	IV	IV	III	I or VII
Toxins				
Leukocidins				
*luk*_*PV*_ *SF*	-	-	-	-
*lukE-lukD*	+	+	+	+
*lukM*	-	-	-	-
Hemolysins				
*hla*, *hlg*, *hlg-v*	+	+	+	+
*hlb* (split)^b^	(+)	(+)	(+)	(+)
Peptide cytolysins				
*psmα*, *hld*	+	+	+	+
Staphylococcus enterotoxins				
*sea*	-	+	+ (8/10)	-
			(1,024–2,048 ng/ml)	
*tst*	+	-	-	-
	(200–400 ng/ml)			
*sec*, *sep*	-	-	-	+
*sek*, s*eq*	+	+	-	-
Exfoliative toxins				
*eta*, *etb*, *etd*	-	-	-	-
Others				
*ssl*	+	+	+	+
*edin*	-	-	-	-
Adhesins				
*c12ag* ^c^	+	+	+	+
*cna*	+	+	-	+
*bbp*	-	-	-	-
ACME (*arcA*)	-	-	-	-
Resistance				
β-lactam				
Imipenem (MIC, μg/ml)	16–64	32	0.125–0.5	0.5
Oxacillin (MIC, μg/ml)	128- ≥256	128	32–64	64
Ampicillin (MIC, μg/ml)	32–64 (18/18)	32 (2/2)	4–8 (7/10)	8
			32 (3/10)^d^	
Non β-lactam				
Aminoglycosides	Gm (17/18)		Gm (3/10)^d^	
	Km (18/18)	Km (2/2)	Km (3/10)^d^	
Tetracyclines	Tc (18/18)	Tc (2/2)		
Macrolides	Em (18/18)	Em (2/2)	Em (2/10)^d^	
Lincosamides	Cli (18/18)	Cli (2/2)	Cli (2/10)^d^	
Quinolones	Lvx (18/18)		Lvx (10/10)	
Rifampicin	Rif (18/18)			
Chloramphenicol	Cp (14/18)^d^		Cp (9/10)^d^	Cp^d^
Sulfamethoxazole	Su (18/18)	Su (2/2)		
Plasmids (kb)	2.9 (14/18)		≥25 (3/10),	4.5
			4.6 (1/10),	
			4.5 (3/10),	
			3.9(1/10),	
			2.9 (7/10),	
			2.5 (1/10),	
			2.4(1/10)	

^a^Gm, gentamicin; Km, kanamycin; Tc, tetracycline; Em, erythromycin; Cli, clindamycin; Lvx, levofloxacin; Rif, rifampicin; Cp, chloramphenicol; Su, sulfamethoxazole; UT, untypeable.

^b^Split *hlb* gene due to insertion of φSa3.

^c^ *c12ag*, core 12 adhesin genes, *icaA*, *icaD*, *eno*, *fnbA*, *fnbB*, *ebpS*, *clfA*, *clfB*, *fib*, *sdrC*, *sdrD*, *and sdrE*.

^d^Resistance specified by a plasmid.

Group A (n = 20) was the ST239 lineage with two types, ST239/*spa*3(t037)/SCC*mec*III.1.1.2 (group A1; 90%, 18/20) and ST239/*spa*3(t037)/SCC*mec*III.1.1.1 (group A2; 10%, 2/20). All strains in group A1 were *tst* ^+^; TSST-1 production levels were similar to those (50–800 ng/ml; average, 313 ng/ml) of the ST5/SCC*mec*II NY/J clone. They were also highly MDR, including levofloxacin (Lvx) and Rif, and many (77.8%, 14/18) carried a Cp^r^ plasmid (pCp^r^). In the PFGE analysis (Fig 1), group A1 comprised several divergent subclusters with a major cluster (type 239α; 44.4%, 8/18). Group A1 MRSA was designated as ST239_Kras_. Three ST239_Kras_ strains (OC3, OC8C, and OC76) caused fatal HAP, and one strain (OC14C) was from a carrier (hospital worker).

The strains in group A2 were *sea* ^+^ (Table 3). Their PFGE patterns were divergent from group A1 (Fig 1). Group A2 was a common ST239 HA-MRSA in Russia, whereas group A2 showed a slightly narrower MDR spectrum (Table 3). Group A (A1 and A2) exhibited a high level of resistance to imipenem and oxacillin (Table 3), which is consistent with the characteristics of HA-MRSA [31].

Group B (n = 10) was the ST8 lineage with the type *spa*1(t008)/SCC*mec*IV.3.1.1(IVc) (Table 3). Most strains (80%, 8/10) were *sea* ^+^, and produced SEA at high levels (Table 3). All strains shared very similar PFGE patterns, with patterns of 8α (major type; n = 7) and 8β (n = 2) and with no more than a three-band difference, indicating the same clone (designated as ST8_Kras_) (Fig 1). ST8_Kras_ (group B) exhibited a low level of resistance to imipenem and oxacillin (Table 3), in agreement with the characteristics of CA-MRSA [31]; however, all strains were Lvx^r^ and most strains (90%, 9/10) carried a Cp^r^ plasmid.

Four ST8_Kras_ strains (OC8, OC22, OC23, and OC59) caused fatal CAP. One ST8_Kras_ strain (OC11) caused fatal HAP, suggesting that ST8_Kras_ even spread in hospitals. The ST8_Kras_ strains associated with fatal pneumonia were all *sea* ^+^. Two ST8_Kras_ strains were isolated from carriers; OC217 was from a student while OC52 was from a hospital worker.

Group C (n = 1, strain OC50) exhibited ST12/*spa*New(t156)/untypable SCC*mec*, showed low imipenem and oxacillin resistance levels, and carried a Cp^r^ plasmid.

Regarding drug resistance (Table 3), all Lvx^r^ ST8 and ST239 carried *gyrA* (Ser84Leu) and *grlA* (Ser80Phe) mutations, and manifested minimum inhibitory concentrations (MICs) of 4–16 μg/ml. Rif^r^ ST239_Kras_ strains carried *rpoB* (His481Asn, Ile527Met) mutations, with MICs of ≥256 μg/ml. All Cp^r^ strains carried the *cat* gene, with MICs of 64 μg/ml. All strains were susceptible to trimethoprim, fusidic acid, vancomycin, teicoplanin, linezolid, and mupirocin.

### Distribution and transfer of drug resistance plasmids

The plasmid data of Krasnoyarsk MRSA are summarized in Table 3, Fig 1, S1 and S2 Figs. Many MRSA strains carried only a small pCp^r^, and the same 2.9-kb pCp^r^ was present in 14 (77.8%) out of 18 ST239_Kras_ strains (group A1) and in seven (70%) out of 10 ST8_Kras_ (group B) strains (Fig 1). This 2.9-kb pCp^r^ was 99.9% homologous to the 2.9-kb pCp^r^ of emerging ST239 MRSA (*spa*351[t030]/SCC*mec*III.1.1.4) from Vladivostok (S1 and S2A-a Figs). One ST8_Kras_ strain carried a 3.9-kb pCp^r^ (Fig 1, S1 and S2A-b Figs). Furthermore, three ST8_Kras_ strains carried the new mosaic 4.5-kb pCp^r^ (Fig 1, S1 and S2A-c Figs); two of these strains carried two distinct species (2.9- and 4.5-kb) of pCp^r^ (Fig 1 and S1 Fig).

Three ST8_Kras_ strains carried large (≥25-kb) antibiotic and antiseptic resistance plasmids (alternatively defined as a penicillinase plasmid, pPCase), in addition to pCp^r^ (Fig 1 and S1 Fig); two of the three ST8_Kras_ strains also carried a small (2.4- or 2.5-kb) pEM^r^ (Fig 1, S1 and S2B Figs).

All plasmids were transferred to *S*. *aureus* RN2677 (recipient) in the bacterial mixed culture at frequencies ranging from 10^-5^ to 10^-7^ (S1D Fig). Tn*554*, carrying the *ermA*, *spc* genes, of ST239_Kras_ strains was also transferred to RN2677 (S1D Fig), most likely through a Tn*554* circular intermediate (S1A and S1C Fig). Of these, the 2.9-kb pCp^r^ exhibited superior transfer frequencies over transmissible (Tra^+^) pPCase (S1D Fig).

### Comparative genomics of ST239_Kras_ (strain OC3)

**Fig 2:**
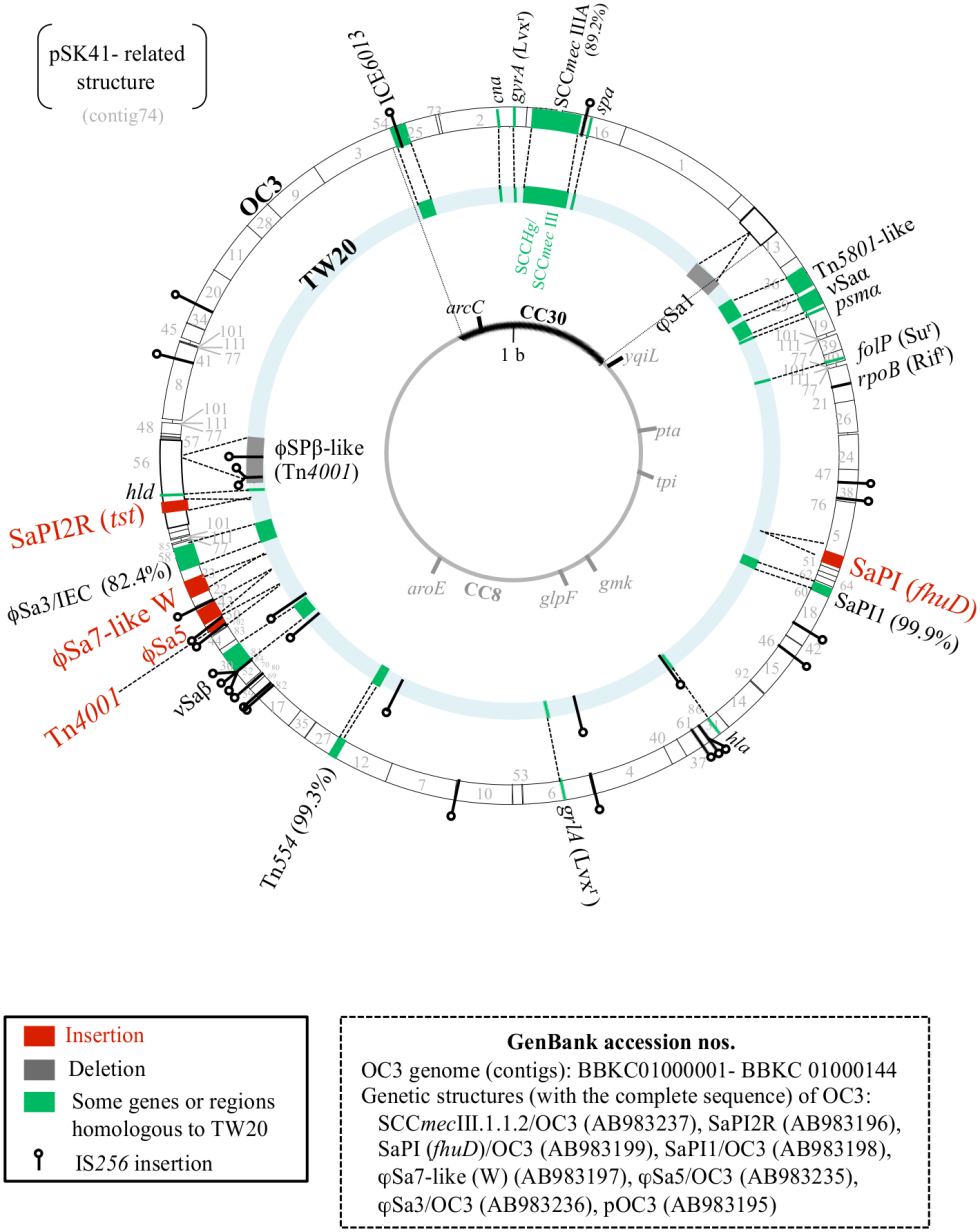
Genome information for the ST239_Kras_ strain OC3, in comparison with the ST239 MRSA strain TW20. The ST239_Kras_ OC3 genome contigs (including filled contigs and complete structures; total 2.91-Mb) were mapped on the 3,043,210-bp TW20 genome (GenBank accession number FN433596); in the figure, the two genome structures were drawn as two circles on a common genome map, outside OC3 and inside TW20. Genome information included staphylococcal cassette chromosome *mec* (SCC*mec*), other drug resistance structures (such as a transposon, Tn, plasmid-related structure, and gene mutations), characteristic virulence genes, phages, *S*. *aureus* pathogenicity islands (SaPIs), genomic islands (νSa), and characteristic insertion sequences (ISs). SCC*mec*: SCC*mec*IIIA (in OC3), SCC*mec*III.1.1.2; SCC*mec*III (in TW20), SCC*mec*III.1.1.1 connected to SCC*Hg*. Drug resistance (gene mutations): Lvx^r^, levofloxacin resistance; Rif^r^, rifampicin resistance; Su^r^, sulfamethoxazole resistance. Virulence genes (region): *tst*, toxic shock syndrome toxin-1 gene; *hld*, δ-hemolysin gene; *cna*, collagen adhesin gene; *spa*, protein A gene; *psmα*, phenol-soluble modulin (PSM) gene; *hla*, α-hemolysin (α-toxin) gene; IEC, immune evasion cluster. The CC30 and CC8 genome sections are from Holden *et al*. [19], and the genetic element IEC*6013* is from [60]. The plasmid pOC3 (2,908 bp; contig 75) of strain OC3 is not shown in the figure. The location of pSK41-related structure (with two IS*431* repeats at both ends) currently remains uncertain.

The OC3 genome was estimated to be at least 2.93-Mb in size, with a 2,908-bp pCp^r^ (pOC3). A total of 2.91-Mb (approximately 99.3% of the determined genome sequences) was mapped on the TW20 genome (Fig 2).

The OC3 genome most likely consisted of two (ST30-like and ST8-like) sections, similar to TW20[19,60]. On the ST30-like section, OC3 carried the collagen-adhesin (Cna) gene (*cna*), IEC*6013* (with the Tn*552* insertion), and the *spa* gene (type3-t037) similar to TW20; however, OC3 lacked phage φSa1, and SCC*mec* was divergent (SCC*mec*III.1.1.2 for OC3 vs. SCC*mec*III.1.1.1 for TW20).

On the ST8-like section, there were five characteristic insertions in the OC3 genome: i) a *tst* ^+^ *S*. *aureus* pathogenicity island (SaPI2R), ii) a completely unique phage (designated as φSa7-like W), iii) a phage, φSa5, iv) a transposon, Tn*4001*, and v) a SaPI carrying the ferrichrome ABC transporter homologue gene (*fhuD*) (designated as SaPI *fhuD*). There were also two characteristic deletions in the OC3 genome: i) a phage, φSPβ-like (carrying the *sasX* gene and Tn*4001*) and ii) the *dfrG* gene in Tn*5801*-like, resulting in a trimethoprim-susceptible phenotype.

A large number of copies of the insertion sequence IS*256* (≥22/genome) were present in OC3, while TW20 only had eight copies. Regarding the MDR of OC3, the 15 genes identified were: i) nine drug resistance genes: *mecA* on SCC*mec*III.1.1.2, *ermA* and *spc* on Tn*554*, *blaZ* on Tn*552*/ICE*6013*, *aacA-aphD* on Tn*4001*, *tetM* on Tn*5801*-like, *cat* on pOC3, and *ble* and *aadD* on a pSK41-related structure; ii) four drug resistance mutations: *gyrA* (S84L) and *grlA* (S80F) for Lvx^r^ [61], *rpoB* (H481N, I527M) for Rif^r^ [62], and *folP* (F17L, V30I, T31N, M37I, I58V, T59S, V60L, L64M, I101M, V117I, V126I; these replacements caused MIC of ≥512 μg/ml and corresponded to 11 out of 13 replacements in strain V2157I [63]) for sulfamethoxazole resistance; and iii) two heavy metal resistance genes: *mer* and *cadA* on SCC*mec*III.1.1.2.

### SCC*mec*III.1.1.2

**Fig 3:**
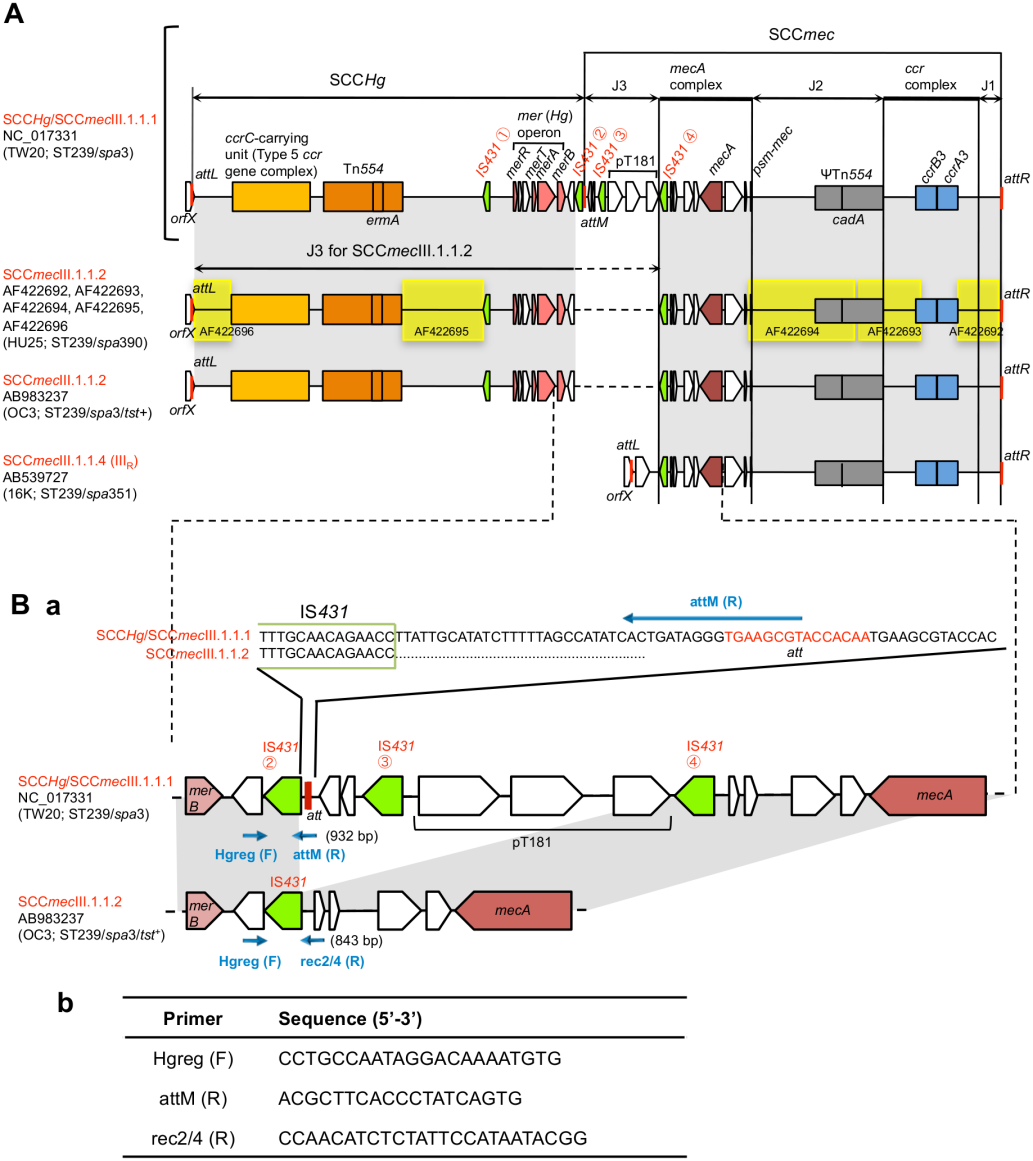
SCC*mec*III.1.1.2 structure of the ST239_Kras_ strain OC3, in comparison with the three structures: SCC*Hg*/SCC*mec*III.1.1.1 of the strain TW20, SCC*mec*III.1.1.2 of the strain HU25, and SCC*mec*III.1.1.4 of the strain 16K. Isolation of ST239 strains: OC3, Krasnoyarsk; TW20, London; HU25, Brazil; 16K, Vladivostok. Homologous regions are shaded in each comparison. In A, when compared with SCC*Hg*/SCC*mec*III.1.1.1 (of TW20), SCC*mec*III.1.1.2 (of OC3 and HU25) lacked the middle IS*431②*-IS*431*④ region. The J3 region of SCC*mec*III.1.1.2 corresponded to the bulk of SCC*Hg*. SCC*mec*III.1.1.4 (of 16K) lacked SCC*Hg*. In B, the primer set Hgreg (F)/attM (R) detected *attM*, and the primer set Hgreg (F)/rec2/4 (R) identified recombination between IS*431* copies ② and ④.

SCC*mec*III.1.1.2 (OC3) was 61,780 bp in size, with 15-bp *att* direct-repeat sequences (*attL*, *attR*). Its structure was identical to that of reference strain HU25 of the Brazilian clone, with ancestral SCC*mec*IIIA, however, markedly distinct from TW20, which had the two-SCC cassette array SCC*Hg*-SCC*mec*III.1.1.1 (35,310 bp), as shown in Fig 3A.

SCC*Hg*-SCC*mec*III.1.1.1 (TW20) had four copies of IS*431* (① to ④) in direct orientation at the boundary region of two SCCs, suggesting a recombination between two IS*431* copies (② and ④) for SCC*mec*III.1.1.2 conversion; the J3 region of SCC*mec*III.1.1.2 (OC3) and SCC*mec*III.1.1.1 (TW20) was, thus, divergent: 33,011 bp vs. 6,266 bp.

To confirm that all ST239_Kras_ strains had the same IS*431* recombination type, the PCR primer sets, Hgreg (F)/attM (R) (to detect *attM*) and Hgreg (F)/rec2/4 (R) (to identify recombination between IS*431* copies ② and ④), were designed (Fig 3B). The results of the PCR assays clearly demonstrated that all ST239_Kras_ strains had the SCC*mec*III.1.1.2 (IIIA) structure of the Brazilian clone. In Russia (Krasnoyarsk and Vladivostok), SCC*mec*III.1.1.4 and SCC*mec*III.1.1.1 had no SCC*Hg* linkage, as shown in Fig 3A.

### ϕSa7-like (W)

**Fig 4:**
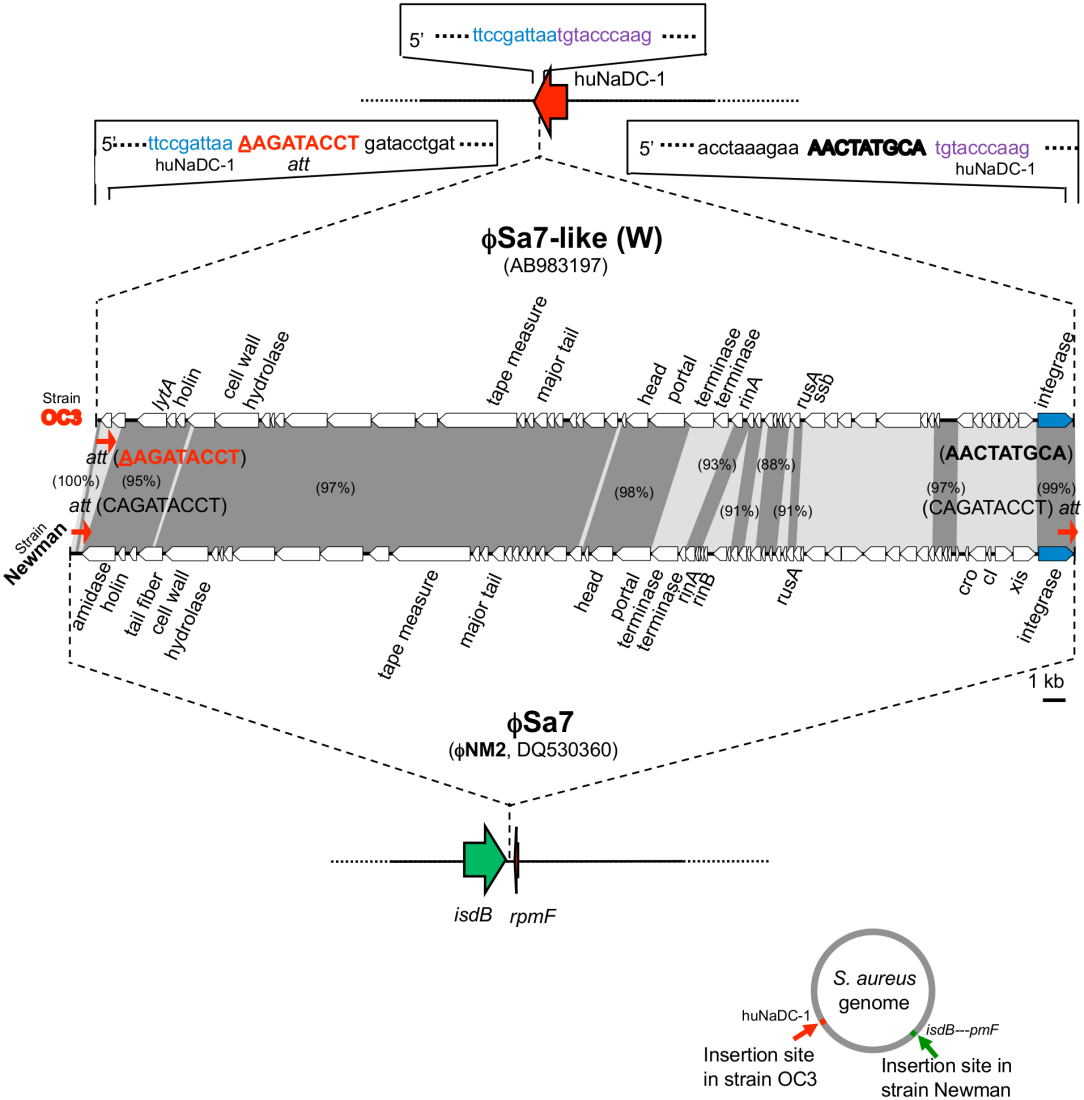
Structure of a phage φSa7-like (W) of the ST239_Kras_ strain OC3. φSa7-like (W) was compared with φSa7 of the strain Newman for the phage structure, the integration site (*att*) sequence, and integration site. Homologous regions are shaded in the comparison. The figure at the lower right side indicates each integration site on the *S*. *aureus* chromosome. The target huNaDC-1 sequence of φSa7-like (W), shown at the top of the figure (in blue and purple), was also present in the huNaDC-1 gene of other *S*. *aureus* strains.

φSa7-like (W) of OC3 was 42,359 bp in size and inserted into the huNaDC-1 gene. As shown in Fig 4, the integrase gene showed high similarity (mostly 100%) to the phage 7 (φSa7) integrase gene of the following strains: NM2 of the strain Newman (Fig 4), ST8 strains (GenBank accession numbers, AP009351.1 and CP007499.1), ST30 strain (GenBank accession number, LN626917.1), ST133 strain (GenBank accession number, CP001996.1), and ST239 strains (GenBank accession numbers, CP005288.1, CP006838.1, CP009681.1), indicating that φSa7-like (W) is a φSa7 family member. φSa7-like (W) had the φSa7 *att*-like 9-bp sequence on the left-side end.

However, the 9-bp right-side sequence of φSa7-like (W) was divergent; there was no φSa7 *att* on the right side (Fig 4). Moreover, the insertion site (huNaDC-1 gene) of φSa7-like (W) was divergent from that of φSa7, which was generally inserted into the intercistronic region between the *isdB* and *rpmF* genes (Fig 4, the figure on the lower right side). φSa7-like (W) only showed 66% overall homology to φSa7 (NM2).

### SaPI2R carrying *tst*

**Fig 5:**
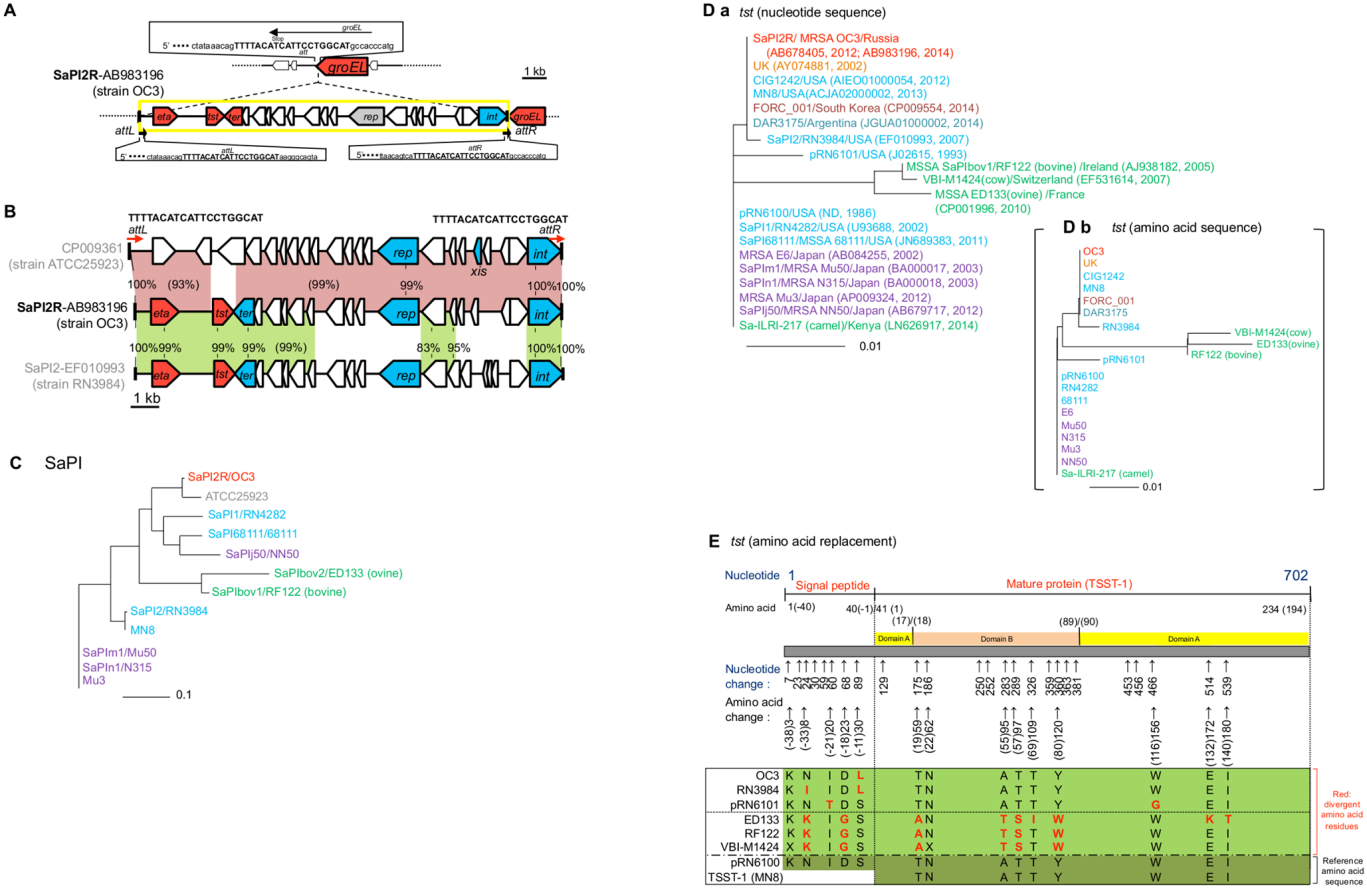
Analysis of the *tst* ^+^ SaPI (SaPI2R) structure and *tst* nucleotide and deduced amino acid sequences of the ST239_Kras_ strain OC3. In A, the integration site (*att*) and *att* sequences of SaPI2R of the ST239_Kras_ strain OC3 are shown. In B, the SaPI2R structure was compared with those of *tst* ^-^ SaPI (ATCC25923) and *tst* ^+^ SaPI2 (RN3984). Homologous regions between SaPI structures are shaded with color. Genes: *tst*, toxic shock syndrome toxin-1 gene; *eta*, *S*. *hyicus* exfoliatin A gene; *ter*, terminase gene (which cleaves multimeric DNA); *rep*, replication initiator gene; *int*, the integrase gene. In C, the nucleotide sequences of *tst* ^+^ SaPIs and *tst* ^-^ SaPI (ATCC25923) were analyzed for phylogenetic diversity. In D-a, the nucleotide sequences of the *tst* genes were analyzed for phylogenetic diversity. In this figure, each GenBank record year is also shown. In D-b, the deduced amino acid sequences of the *tst* gene products were analyzed for phylogenetic diversity. The origin (reported source) of each isolate is indicated by the color of the isolate name: red, Russia; yellow, United Kingdom (UK); blue, United States (USA); dark red, Korea; light blue, Argentine; purple, Japan; green, those for animal isolates. In C and D, the scale bar represents substitutions per single-nucleotide polymorphism site. In E, the representative *tst* gene sequences were compared with the reference sequences (of pRN6100). Arrows indicate the positions of the nucleotide and amino acid changes for the representative *tst* genes. At the bottom of the figure (green), different amino acids from the amino acid sequences of purified TSST-1 (MN8; GenBank accession number EFH95768) and the deduced amino acid sequence of the *tst* gene (pRN6100) are indicated in red letters.

SaPI2R (OC3) was 14,819 bp in size, flanked by directly repeated 20-bp *att* sequences (*attL* and *attR*), and inserted into the *groEL* gene (Fig 5A). SaPI2R exhibited high homology (91%) to the *tst* ^-^ SaPI2 of strain ATCC25923, and the 6,810-bp left-side *tst* ^+^ region of SaPI2R showed high homology (99%) to that of *tst* ^+^ SaPI2 (strain RN3984) (Fig 5B). Although SaPI2R was closely related to *tst* ^-^ SaPI2 (ATCC25923), SaPI2R markedly diverged from other *tst* ^+^ SaPIs (Fig 5C).

In the *tst* gene sequence comparison (Fig 5D-a), seven clusters were detected: i) cluster consisting of *tst* from Russia (OC3), UK, USA, South Korea, and Argentine; ii) *tst* cluster from USA (RN3984); iii) *tst* cluster from USA (pRN6101); iv) cluster consisting of *tst* from USA (including pRN6100), Japan (ST5/SCC*mec*II HA-MRSA, NY/J clone; and ST8/SCC*mec*IVl CA-MRSA, ST8 CA-MRSA/J clone), and Kenya (camel strain); and v-vii) three *tst* clusters from Ireland, Switzerland, and France (cow/bovine and ovine strains). The analysis at the deduced amino acid sequence levels produced very similar results (Fig 5D-b).

Russian (OC3) and UK TSST-1 precursors shared the same amino acid sequence with the same one amino acid replacement in the signal peptide region (S-11L; S→L at position -11), when compared with purified TSST-1 protein (MN8) or the precursor protein, deduced from the first USA *tst* gene (pRN6100) (Fig 5E). Regarding the *tst* genes from clinical isolates, amino acid replacements in the mature toxin (TSST-1) region were very rare, in contrast to the *tst* genes from animal isolates with distinct host specificity (Fig 5E). We were unable to determine why the *tst* gene from camel (Kenya) had the clinical type sequence.

### SaPI1 carrying *sek* and *seq*

SaPI1 (OC3) was 14,577 bp in size (with 17-bp *att* at both ends), carried the superantigen (SE) genes (*sek* and *seq*), and was inserted into a non-coding region. SaPI1 (OC3) was highly homologous (99.9%) to SaPI1 (TW20) (S3A Fig). The SEK and SEQ amino acid sequences were the same between OC3 and TW20, with the unique amino acid replacement F119L (S3B-a Fig) and two unique amino acid replacements D194N and T201A (which corresponded to two out of seven replacements in USA300 SaPI5) (S3B-b Fig), respectively.

### ϕSa3 carrying immune evasion genes

φSa3 (OC3) was 43,681 bp in size (with 13-bp *att* at both ends) and was inserted into the *hlb* gene. φSa3 (OC3) showed 76% homology to that of strain CN1, sharing the same *att* and same integration site (S4 Fig). φSa3 (OC3) had the immune evasion cluster (IEC) on the left-end side, with the immune evasion genes *sak* (for staphylokinase, SAK) and *scn* (for staphylococcal complement inhibitor, SCIN), but lacked *chp* (for chemotaxis inhibitory protein of *S*. *aureus*, CHIPS) present in CN1 (S4 Fig). The IEC region of TW20 and ST8_Kras_ strain OC8 showed 99.3% homology and carried *sea*, in addition to *sak*, and *scn* (S4 Fig).

### Other relevant genetic structures

Tn*4001* (OC3), flanked by two IS*256*, was 6,483 bp in size and inserted into the noncoding region, located downstream of the ThiJ/PfpI family protein gene (Fig 2); in TW20, Tn*4001* was present within φSPβ-like.

The pSK41-related resistance structure, flanked by two IS*431*, was 4,039 bp in size and carried the two drug resistance genes *ble* and *aadD* (Fig 2); its location on the genome currently remains unknown.

SaPI (*fhuD*) was 15,756 bp in size and inserted into the noncoding region, located downstream of the SsrA-binding protein gene. It showed only 69% overall homology to *fhuD* ^+^ SaPIm4 from the NY/J clone (Mu50). The 10.2-kb left-side half exhibited high homology (95%) to *fhuD* ^-^ SaPIj50 from Japanese ST8/SCC*mec*IVl CA-MRSA, suggesting that SaPI (*fhuD*) is a new mosaic SaPI (S5 Fig).

φSa5 (OC3), with 10-bp *att* at both ends, was 44,424 bp in size and inserted into the hypothetical gene (for protein AGY89988.1), located downstream of the ThiJ/PfpI family protein gene. It was the most similar to φSa5 (XN108), albeit with only 63% homology (S6 Fig); φSa5 (OC3) is a new mosaic phage.

### Elevated mRNA expression of cytolytic peptide genes in ST239_Kras_

**Fig 6:**
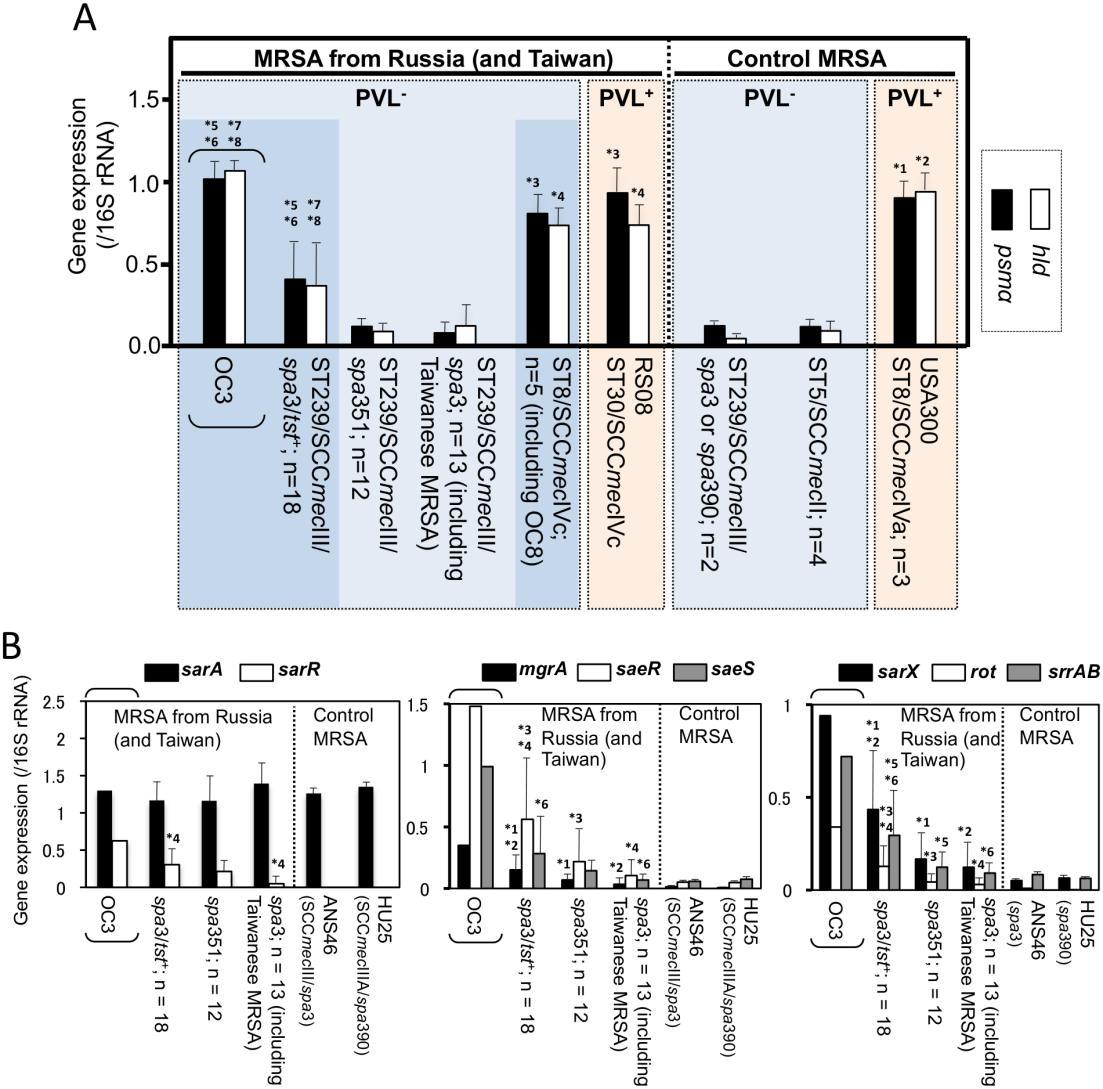
mRNA expression levels of cytolytic peptide genes (*psmα* and *hld*) in ST239_Kras_ and ST8_Kras_ strains (A) and of regulatory genes in ST239_Kras_ strains (B), in comparison with CA- and HA-MRSA reference strains and other ST239 MRSA strains. In A, in the right-side control MRSA box, CA-MRSA, which shows high expression levels, is marked in red; and HA-MRSA, which shows low expression levels, is marked in light blue. In the left-side MRSA box, CA-MRSA (strain RS08), which showed high expression levels as expected, is marked in red; CA-MRSA ST8_Kras_ (ST8/SCC*mec*IVc) strains, including OC8, also showed high expression levels (dark blue box on the right side). ST239 HA-MRSA strains, which showed low expression levels as expected, are marked in light blue. However, ST239_Kras_ (ST239/SCC*mec*III/*spa*3/*tst* ^+^), including OC3, unexpectedly showed high expression levels, similar to CA-MRSA; this box is marked in dark blue on the left side. Regarding *psmα*: *1, *P* < 0.05 vs. control ST239/SCC*mec*III and ST5/SCC*mec*II; *3, *P* < 0.05 vs. control ST239/SCC*mec*III and ST5/SCC*mec*II; *5, *P* < 0.05 vs. control ST239/SCC*mec*III and ST5/SCC*mec*II; *6, *P* < 0.05 vs. Russian ST239/SCC*mec*III/*spa*3 and *spa*351. Regarding *hld*: *2, *P* < 0.05 vs. control ST239/SCC*mec*III and ST5/SCC*mec*II; *4, *P* < 0.05 vs. control ST239/SCC*mec*III and ST5/SCC*mec*II; *7, *P* < 0.05 vs. control ST239/SCC*mec*III and ST5/SCC*mec*II; *8, *P* < 0.05 vs. Russian ST239/SCC*mec*III/*spa*3 and *spa*351. In B, the *psmα* expression levels of ST239 MRSA strains were examined. *spa*3/*tst* ^+^, ST239_Kras_. No significant difference was observed between ST239_Kras_ and other ST239 MRSA for *sarA* gene expression. *1, *P* < 0.05 vs. *spa*351; *2, *P* < 0.05 vs. *spa*3; *3, *P* < 0.05 vs. *spa*351; *4, *P* < 0.05 vs. *spa*3; *5, *P* < 0.05 vs. *spa*351; *6, *P* < 0.05 vs. *spa*3.

ST239_Kras_, including strain OC3, expressed the *psmα* and *hld* genes at high levels, similar to CA-MRSA USA300 and Russian CA-MRSA (RS08 and ST8_Kras_), but significantly higher than HA-MRSA, ST5/SCC*mec*II (the NY/J clone) and other ST239/SCC*mec*III, including reference strains HU25 and ANS46 (*P* <0.05), as shown in Fig 6A.

Regarding the expression levels of transcriptional regulatory genes (Fig 6B), although no significant difference was observed among the ST239 strains for *sarA*, ST239_Kras_, including OC3, showed higher levels of expression for *sarR*, *mgrA*, *saeR*, *saeS*, *sarX*, *rot*, and *srrAB* (*P* <0.05), compared with the other ST239 strains, including reference strains HU25 and ASN46.

### Comparison of ST239_Kras_ and ST239 MRSA from other regions of Russia

The emerging HA-MRSA with the genotype ST239/*spa*351(t030)/SCC*mec*III.1.1.4 was detected in the European region (Moscow and St. Petersburg), Ural region (Kurgan), and Far Eastern region (Vladivostok) (S1 Table), except for the Siberian region (Krasnoyarsk) (Table 3). Its *spa* variant, *spa*New(t632), was distributed to the European region (St. Petersburg). ST239_Kras_ was only distributed to the Siberian region (Krasnoyarsk).

Three major divergent clusters were detected in the PFGE analysis (S7 Fig): a large *spa*351/SCC*mec*III.1.1.4 cluster, associated with the Far Eastern region; a large *spa*351-New(t632)/SCC*mec*III.1.1.4 cluster, mainly associated with the European Russia/Ural mountain region (and also the Far Eastern region); and a large *spa*3/SCC*mec*III.1.1.1-III.1.1.2 cluster, associated with the Siberian region and Far Eastern region. The emerging ST239/*spa*351(t030)-*spa*New(t632)/SCC*mec*III.1.1.4 type appeared to be slightly divergent between the European/Ural region and Far Eastern region. ST239_Kras_ was divergent from those emerging types, and comprised *spa*3 subclusters with reference strains ASN46 and HU25.

## Discussion

Regarding MRSA epidemiology in Russia, the incidence of MRSA (among *S*. *aureus* isolates) was 0–89.5% (average, 33.5%) in 2000 [64], 18% in 2004 [65], 54.4% in 2006–2008 [66], 32.1% in 2007 and 16.6% in 2012 [67]. The dominant MRSA types in Russia are ST239/*spa*3(t037)/SCC*mec*III, followed by ST239/*spa*351(t030)/SCC*mec*III and ST8/*spa*1 (t008)/SCC*mec*IV [49]. Russia is geographically classified into three major regions: European, Siberian, and Far Eastern. The present study described for the first time MRSA and its invasive infection in the Siberian region. In Krasnoyarsk (2007–2011), the incidence of HA-MRSA was at similar levels, while the incidence of CA-MRSA was a little bit lower.

However, regarding MRSA types, the most prevalent HA-MRSA was ST239_Kras_, a novel regional variant of the ST239 lineage (S2 Table, S8 Fig), in contrast to the European (Moscow and St. Petersburg), Ural (Kurgan), and Far Eastern (Vladivostok) regions, where the emerging ST239/*spa*351(t030)/SCC*mec*III.1.1.4 type has recently become common [52]. ST239_Kras_ is highly-virulent HA-MRSA with fatal HAP cases (with bacteremia) being reported. The ages of patients with fatal HAP were consistent with previous HA-MRSA data [34]. The genetic divergence in PFGE patterns strongly suggested that ST239_Kras_ infections persisted and spread among patients and carriers (hospital workers) at least since 2007.

Regarding the ST8 lineage, a single unique MDR MRSA clone (ST8_Kras_) with very similar PFGE patterns had persisted and spread. The *spa* type (*spa*1-t008) was the same as previous Russian ST8 [49], but divergent from that (*spa*826; t, unknown) of Vladivostok ST8 [52]. ST8_Kras_ was Lvx^r^ with the same mutations as HA-MRSA ST239_Kras_. ST8_Kras_ was a successful CA-MRSA, with not only fatal CAP, but also fatal HAP cases (with bacteremia). The fatal CAP cases included one infant and one young child death, consistent with previous CA-MRSA infections [34]. ST8_Kras_ carriers were also identified, suggesting its potential to be become widespread.

PVL^+^ CA-MRSA, such as ST8 USA300 [12,23,68], has become a major public concern [3,5,12, 23,24,34,68,69]; however, MRSA invasive infections occurred regardless of PVL^+^ or PVL^-^ [70–72]. In Russia, MRSA has mostly been PVL^-^ [49,65,67], with only two PVL^+^ cases [50,73]. In Krasnoyarsk, all MRSA were PVL^-^, albeit with PVL^+^ methicillin-susceptible *S*. *aureus* cases (at around 10%) associated with pyogenic skin infections (such as furuncles).

In the present study, we also focused onto the MVFs of MRSA. The hyper virulence of CA-MRSA USA300 has been attributed to MVFs, such as PVL, ACME-related factors, α-hemolysin (Hla), the elevated production of PSMs, and SEK (*sek2*) and SEQ (*seq2*) [37,68].

Regarding ST239_Kras_, a unique set of MVFs included TSST-1, the elevated expression of PSMα/Hld, Hla, SEK/SEQ, SCIN/SAK, and Cna. Of those factors, TSST-1 has been associated with toxic shock syndrome (TSS) [74–76] and neonatal TSS-like exanthematous disease [77] through a cytokine storm [78–82], is associated with invasive endocarditis [83], and is an immune evasion factor [84]. In Japan, major HA-MRSA and CA-MRSA are both *tst* ^+^, and associated with invasive infections, including pneumonia and bacteremia [21,71,85].

ST239_Kras_ strongly expressed PSMα (and Hld), a common characteristic of CA-MRSA [12,40], which is cytolytic against human cells [40] and possibly associated with bacteremia and abscesses [12] as well as the establishment of an MRSA niche [86]. Community infection (including necrotizing pneumonia) or colonization from HA-MRSA that strongly expresses PSMα/Hld includes ST5/SCC*mec*II (NY/J) [72] and ST764/SCC*mec*II cases [56].

In ST239_Kras_, some transcriptional regulatory genes, except for *sarA* [87,88], were also up-regulated. They included transcriptional-positive regulators such as *mgrA* [89], *saeR/S* [89], and *sarX* [90]; and transcriptional-negative regulators such as *sarR* [87,88], *rot* [43], and *srrAB* [91]. Super IS*256* copies in ST239_Kras_ may be responsible for these transcriptional regulations, as has been reported with *S*. *epidermidis* [92] or *rot* [43]. IS*256* also contributes to Tn*4001* [93].

The acquisition of *sek* and *seq* with synonymous substitutions (*sek2*, *seq2*) may partly explain the hyper virulence of USA300 [37]. ST239_Kras_ (OC3) shared the same (unique) SEK and SEQ sequences with TW20, which were distinct from those of USA300.

Immune evasion factor genes are generally clustered in the IEC locus in φSa3 [44,94], but are often detected in φSa7 [94] or νSaβ [29]. ST239_Kras_ carried two those genes, *sak* and *scn*, in IEC, while USA300 carried three genes *sak*, *chp*, and *scn* (GenBank accession no. CP000255). TW20 and ST8_Kras_ (OC8) carried a distinct set of three genes, *sak*, *scn*, and *sea*.

Cna is a cell wall-associated adhesin [95,96], and associated with pneumonia [97] and bullous impetigo [98]. Cna is also a potential immune evasion factor [99]. Taken together, ST239_Kras_’ MVFs include professional factors for adherence, immune evasion, and specific lesions and symptoms.

ST8_Kras_ possessed a distinct set of MVFs, which included SEA, the strong expression of PSMα/Hld, SAK/SCIN, and Hla. Of these factors, SEA is associated with the severity of infections (sepsis and shock) [100] and promotes bacterial survival *in vivo* [101]. ST8_Kras_ has attracted attention because of its high mortality rate for MRSA CAP, including pediatric deaths. The whole genome of ST8_Kras_ is now being investigated to further characterize ST8_Kras_’ MVFs.

Discussion on the evolution of MRSA is also the important points of the present research. Regarding the ST239/SCC*mec*III lineage, this global HA-MRSA [10,20,49,52,102–113,114]) consists of more than five MRSA clades [20]. Historically, the Brazilian clone carried “SCC*mec*IIIA” [9,15,20], while the Hungarian clone carried “SCC*mec*III” [17]. “SCC*mec*IIIA” is now one large fused SCC (SCC*mec*III.1.1.2), derived from two SCC-linked “SCC*mec*III” through IS*431*-recombination [8,9,115–117]. ST239_Kras_ had the same IS*431*-recombination type as that of the Brazilian clone. In Russia, no SCC*Hg*-SCC*mec*III link was present [52].

Moreover, ST239_Kras_ carried the *tst* gene on SaPI [42,118], for the first time in the ST239 lineage. The same *tst* gene was present in the United Kingdom before the isolation of ST239_Kras_, suggesting the potential salvage of *tst* in Europe. ST239_Kras_ also carried a completely unique, domestic phage, φSa7-like (W). Phages [19,20,44,119–121] are a possible tool for *S*. *aureus* diversification, and classified according to the integrase gene types [120]. φSa7-like (W) was classified as integrase type 7 (Sa7*int*); however, φSa7-like (W) had no repeats of the terminal *att* sequence, similar to Tn*554* [52,122–124], and had a unique insertion site distinct from φSa7. In addition, ST239_Kras_ exhibited the characteristics of CA-MRSA, i.e., the strong expression of the cytolytic peptide gene (as described above).

Regarding ST239 MRSA transmission, the Brazilian clone spread to Portugal [9,46], Central Europe (Germany, Poland, and Czech Republic), Northern Europe (Finland) [17], and Eastern Europe/West Asia (Georgia) [125]. Krasnoyarsk has had a historically close relationship to the European region (St. Petersburg and Moscow). The (Southeast) Asian clade, including London strain TW20, which was likely transmitted from Southeast Asia [2,20], carried characteristic φSPβ-like (S2 Table) [19,20,126–128], while ST239_Kras_ lacked φSPβ-like. Based on these findings and our results, we herein proposed a new Russian clade (representative strain, OC3) in the ST239/SCC*mec*III lineage, and also speculated that ST239_Kras_ originated in the Brazilian clone, with the possible transmission route of Brazil-Europe (West-Central-North/East)-Russia (European-Siberian) (S9 Fig). Further genome-level analysis is needed for the understanding of evolution.

The plasmid distribution in Krasnoyarsk was unique. Many MRSA only carried pCp^r^ and often carried two pCp^r^ species, in contrast to some other country’s cases with no pCp^r^ [19,23,28,29,71,129,130], or Vladivostok’s cases with multiple plasmids [52]. In Russia, inexpensive Cp is commonly administered to patients without a doctor’s prescription as an ointment for skin injuries or burns, as a tablet for gastroenteritis, and as an eye lotion, providing MRSA with strong pressure to carry a pCp^r^. A small (2.9-kb) pCp^r^ must be transferred, even in nature, possibly through the rolling circle (RC) manner of replication [41,131,132], similar to the replication that occurs during the conjugation of large Tra^+^ plasmids [1,41,132–134]. pEM^r^ [135] and Tn*554*, with a circular intermediate [52,136], may follow pCp^r^-like transfer.

In conclusion, we identified novel regional variants of the ST239 and ST8 lineages (ST239_Kras_ and ST8_Kras_), in Siberian Russia (Krasnoyarsk), in which international research had never previously focused on MRSA and its invasive infections. ST239_Kras_ and ST8_Kras_ were MDR and had clonally (albeit with divergence) and widely spread, with fatal cases of HAP and CAP with bacteremia. The 15-day mortality rate for MRSA CAP was significantly higher than that for MRSA HAP, and fatal cases of ST8_Kras_ CAP included infant and young child deaths. According to the recent accumulation of information showing that successful MRSA, associated with large epidemics, has a unique set of MVFs, we speculated that fatal cases of ST239_Kras_ HAP were caused by the unique combination of TSST-1, the strong expression of PSMα/Hld, Hla, SEK/SEQ, SAK/SCIN, and Cna, while fatal cases of ST8_Kras_ CAP were attributed to the combination of SEA, the strong expression of PSMα/Hld, Hla, and SAK/SCIN. ST239_Kras_ carried a completely unique phage and mobile DNA, and exhibited unique virulence phenotypes; therefore, ST239_Kras_ represented a new (Siberian Russian) clade of the ST239 lineage, which was created through regional stepwise evolution during its possible Brazil-Europe-Russia transmission. Small resistance plasmids spread widely enough to not be ignored and in a unique manner among MRSA.

## Supporting Information

S1 Fig Plasmid analysis (A to C) and plasmid transfer in a mixed bacterial culture (D) of MRSA from Krasnoyarsk, in comparison with the ST239 MRSA strain 16K from Vladivostok. In A; RN, RN2677 (recipient). Covalently closed circular (CCC) plasmid DNA, isolated from MRSA and transconjugants (RN2677 carrying plasmids), was electrophoresed in 1% agarose. Plasmid sizes were determined using reference plasmids with known molecular sizes. Plasmids (color): Cp^r^ (yellow), chloramphenicol resistance plasmid; Em^r^ (blue), erythromycin resistance plasmid; Gm^r^ (red), gentamicin resistance plasmid. Regarding plasmids marked with *, the entire plasmid sequence was determined. In B-a, CCC plasmid DNA was electrophoresed in 0.6% agarose. RN, RN2677. In B-b, CCC plasmid DNA was digested with *Eco*RI, and the digests were electrophoresed in 0.5% agarose. Marker 1, 2.5 kb DNA Ladder; marker 2, λ-*Hin*dIII digest. In C; RN, RN2677. The Tn*554* circular intermediate was detected by PCR; the ST239_Kras_ strain OC3 (lane 2) and Em^r^ transconjugant (Em^r^ RN2677, lane 3) produced positive results (carried the Tn*554* circular intermediate), while RN2677 (lane 4) had no such structure. In D, bacterial mating between MRSA (plasmid-donor) and RN2677 (recipient) was performed by filter mating and non-filter mating methods. Nov, novobiocin; Cp, chloramphenicol; Em, erythromycin; Gm, gentamicin; Cli, clindamycin; Spc, spectinomycin; Amp, ampicillin; Cd, cadmium; EtBr, ethidium bromide; Acr, acriflavin. Transfer frequency, plasmid-positive (drug-resistant) transconjugants/donor.
(TIFF)

S2 Fig Structure analysis of small plasmids specifying for chloramphenicol resistance (A-a to c) and erythromycin/clindamycin resistance (B). Plasmid sequence data were from the GenBank accession numbers described. Homologous regions are shaded in each comparison. Genes: *cap*, chloramphenicol resistance; *rep*, replication initiator protein; *pre*, pre protein; *rlx*, RLX protein; *repL*, replication initiator protein L. Em/Cli^r^, constitutive resistance to erythromycin and clindamycin; Em/Cli^ind^, inducible resistance to erythromycin and clindamycin (due to the presence of the leader peptide sequence in the promoter region upstream of *ermC*).
(TIFF)

S3 Fig Analysis of the *sek* ^+^ *seq* ^+^ SaPI1 structure of the ST239_Kras_ strain OC3. In A, SaPI1 (OC3) showed the highest homology to SaPI1 (TW20). SaPI1 (OC3) was also compared with SaPI5 (USA300). Homologous regions between the SaPI structures are shaded with color. *ear*, penicillin-binding protein fragment. In B, the deduced amino acid sequences of the *sek* and *seq* genes (of OC3, TW20, and USA300) were compared with those of COL. Arrows indicate the positions of the amino acid changes. Different amino acids from the amino acid sequences of COL are indicated in red letters.
(TIFF)

S4 Fig Structure of φSa3 of the ST239_Kras_ strain OC3. φSa3 (OC3) exhibited the highest homology to φSa3 (CN1). The left-side immune evasion cluster (IEC) region was also compared with those of φSa3 (TW20) and ST8_Kras_ strain OC8. Homologous regions are shaded in each comparison. Genes in IEC: *scn*, staphylococcal complement inhibitor (SCIN) gene; *chp*, chemotaxis inhibitory protein of *S*. *aureus* (CHIPS) gene; *sak*, staphylokinase (SAK) gene; sea, staphylococcal enterotoxin A (ETA) gene. The IEC region, carrying *scn* and *sak*, of OC3 (a region from *attL* to *sak*) was 3,541 bp in size, and showed 99% homology to the corresponding region of TW20. The IEC region, carrying *scn*, *sak*, and *sea*, of OC8 (a region from *attL* to *sea*) was 6,022 bp in size, and showed 99.3% homology to the corresponding region of TW20.
(TIFF)

S5 Fig *fhuD* ^+^ SaPI structure of the ST239_Kras_ strain OC3. *fhuD* ^+^ SaPI (OC3) showed only limited homology to any previous SaPI, suggesting a novel mosaic SaPI (*fhuD*). Homologous regions are shaded in each comparison. Genes: *int*, integrase gene; *xis*, excisionase; *rep*, replication initiator gene; *ter*, terminase gene (which cleaves multimeric DNA); *fhuD*, ferrichrome ABC transporter homologue.
(TIFF)

S6 Fig Structure of φSa5 of the ST239_Kras_ strain OC3. φSa5 (OC3) exhibited the highest (but limited) homology to φSa5 (XN108), suggesting a new mosaic phage. Homologous regions are shaded in each comparison.
(TIFF)

S7 Fig Pulsed-field gel electrophoresis (PFGE) analysis of ST239 MRSA strains isolated from the European region (Moscow, St. Petersburg), Ural region (Kurgan), and Far Eastern region (Vladivostok), in comparison with ST239_Kras_. In the dendrogram (left side), a large *spa*3/SCC*mec*III.1.1.1-III.1.1.2 cluster, associated with the Siberian region and Far Eastern region, is shadowed. In the middle of the figure, each Russian region is distinguished by color: red, Siberian region (Krasnoyarsk); green, Far Eastern region (Vladivostok); brown, European region (Moscow, St. Petersburg), purple, Ural region (Kurgan). Reference strains (HU25 and ANS46) are not marked. Regarding PFGE patterns (right side), the PFGE types of ST239 MRSA from Krasnoyarsk are those shown in Fig 1.
(TIFF)

S8 Fig Comparison of SCC*mec*III structures of the whole genome-analyzed ST239 MRSA strains. Eight whole genome-analyzed ST239 strains, shown in this figure, are those described in S2 Table. SCC*mec*III structures were analyzed as shown in Fig 3. Homologous regions are shaded. SCC*mec*III.1.1.new-a and SCC*mec*III.1.1.new-b, SCC*mec*III.1.1 with new J3 regions.
(TIFF)

S9 Fig A proposed model for the regional stepwise evolution of ST239_Kras_, which emerged in Siberian Russia (Krasnoyarsk). ST239_Kras_ is characterized by SCC*mec*III.1.1.2, *tst*, and φSa7-like (W). This figure indicates a possible Brazil-Europe-Russia transmission route for ST239_Kras_, in addition to the territories of some other prevalent ST239 MRSA.
(TIFF)

S1 Table Molecular characterization of ST239 MRSA strains isolated from Moscow, St. Petersburg, Kurgan, and Vladivostok^a^. (XLS)

S2 Table Comparison of the characteristic genetic structures on the ST239 MRSA whole genomes: virulence, drug resistance, and evolution. (XLSX)
