# Supplementary figures and images for: Healthcare- and Community-Associated Methicillin-Resistant Staphylococcus aureus (MRSA) and Fatal Pneumonia with Pediatric Deaths in Krasnoyarsk, Siberian Russia: Unique MRSA's Multiple Virulence Factors, Genome, and Stepwise Evolution
Source: PLoS One. 2015 Jun 5;10(6):e0128017. doi: 10.1371/journal.pone.0128017 (PMC4457420; doi:10.1371/journal.pone.0128017)

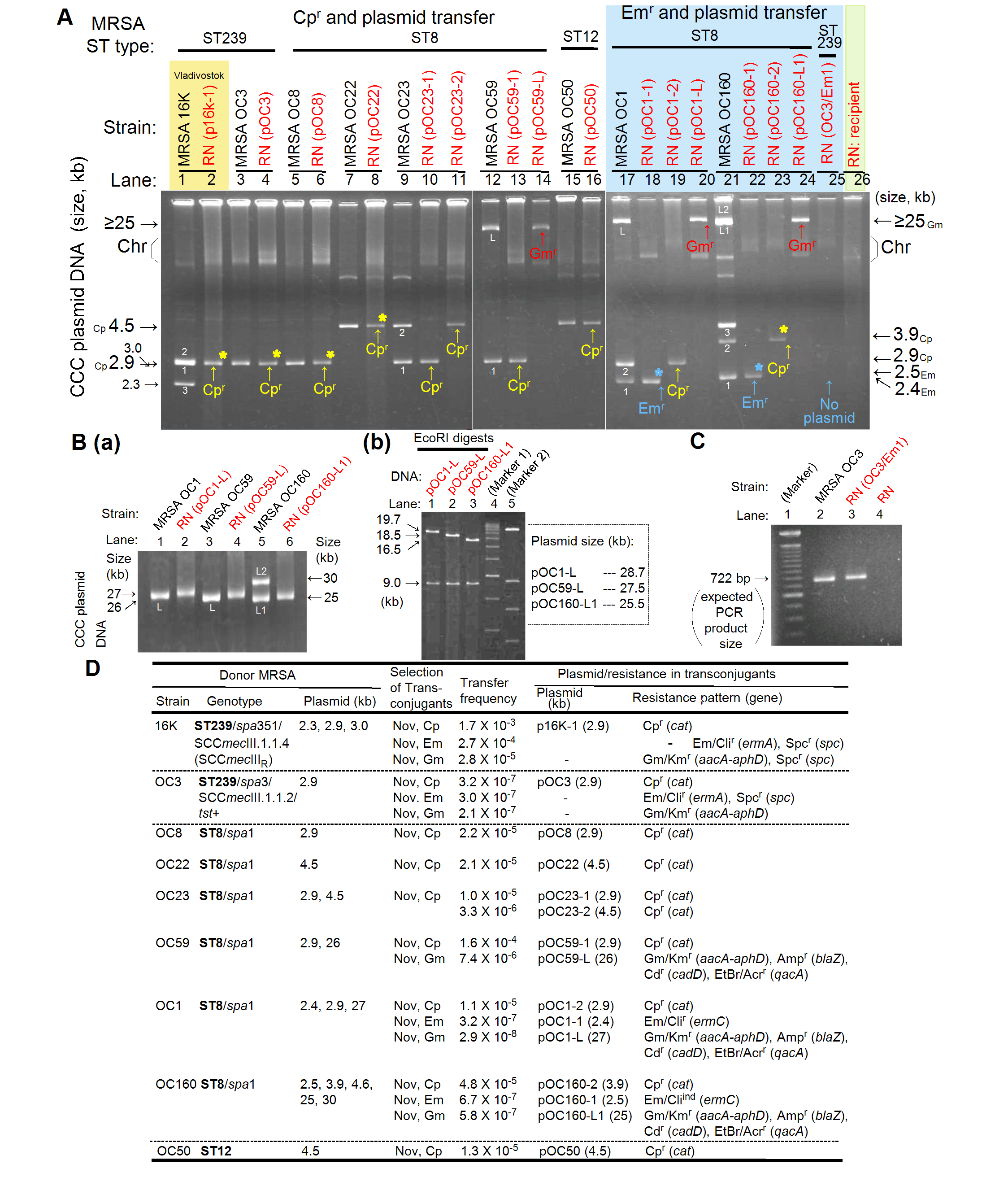

Supplement: S1 Fig — In A; RN, RN2677 (recipient). Covalently closed circular (CCC) plasmid DNA, isolated from MRSA and transconjugants (RN2677 carrying plasmids), was electrophoresed in 1% agarose. Plasmid sizes were determined using reference plasmids with known molecular sizes. Plasmids (color): Cpr (yellow), chloramphenicol resistance plasmid; Emr (blue), erythromycin resistance plasmid; Gmr (red), gentamicin resistance plasmid. Regarding plasmids marked with *, the entire plasmid sequence was determined. In B-a, CCC plasmid DNA was electrophoresed in 0.6% agarose. RN, RN2677. In B-b, CCC plasmid DNA was digested with EcoRI, and the digests were electrophoresed in 0.5% agarose. Marker 1, 2.5 kb DNA Ladder; marker 2, λ-HindIII digest. In C; RN, RN2677. The Tn554 circular intermediate was detected by PCR; the ST239Kras strain OC3 (lane 2) and Emr transconjugant (Emr RN2677, lane 3) produced positive results (carried the Tn554 circular intermediate), while RN2677 (lane 4) had no such structure. In D, bacterial mating between MRSA (plasmid-donor) and RN2677 (recipient) was performed by filter mating and non-filter mating methods. Nov, novobiocin; Cp, chloramphenicol; Em, erythromycin; Gm, gentamicin; Cli, clindamycin; Spc, spectinomycin; Amp, ampicillin; Cd, cadmium; EtBr, ethidium bromide; Acr, acriflavin. Transfer frequency, plasmid-positive (drug-resistant) transconjugants/donor. (TIFF) [file pone.0128017.s001.tiff]

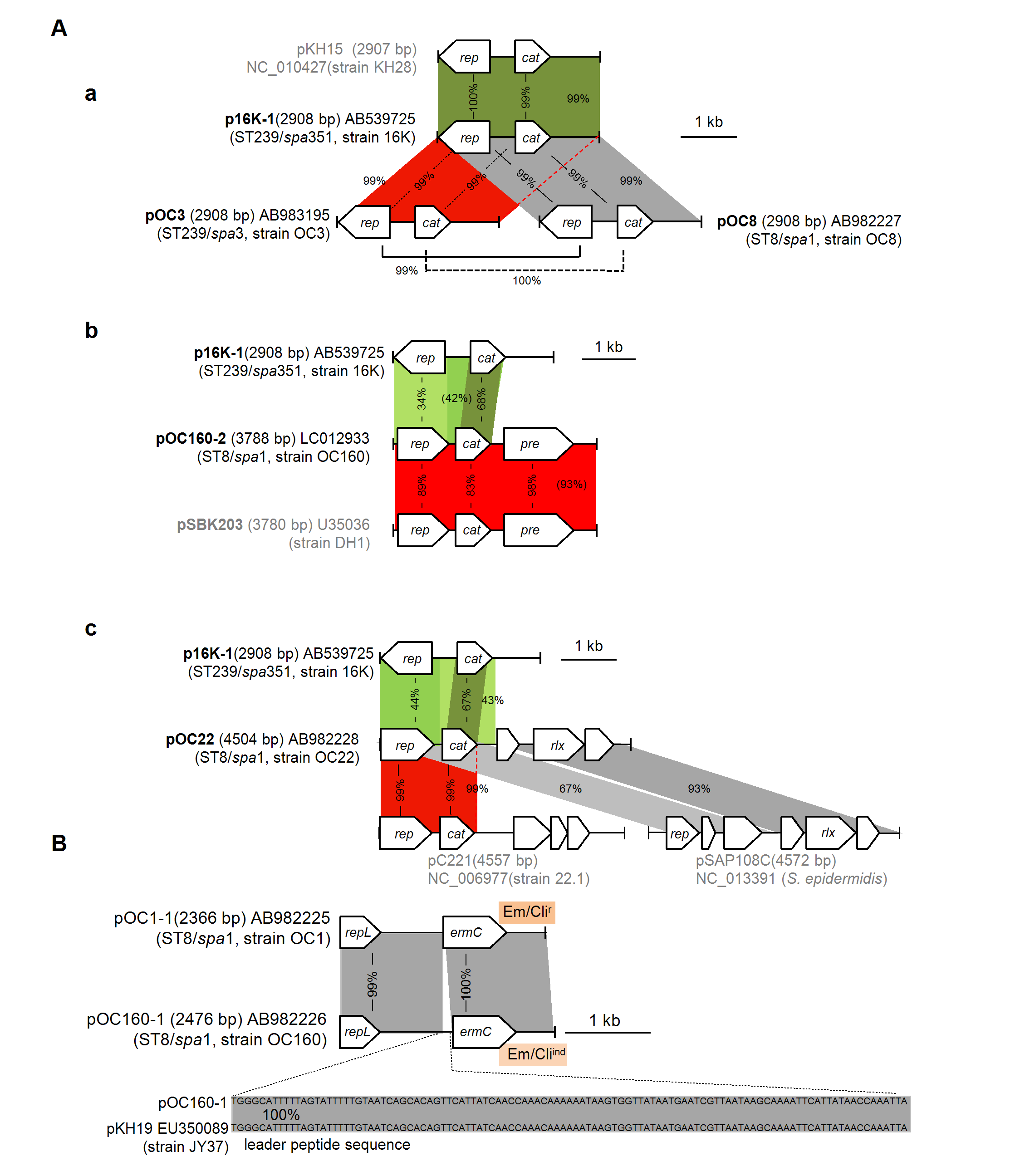

Supplement: S2 Fig — Plasmid sequence data were from the GenBank accession numbers described. Homologous regions are shaded in each comparison. Genes: cap, chloramphenicol resistance; rep, replication initiator protein; pre, pre protein; rlx, RLX protein; repL, replication initiator protein L. Em/Clir, constitutive resistance to erythromycin and clindamycin; Em/Cliind, inducible resistance to erythromycin and clindamycin (due to the presence of the leader peptide sequence in the promoter region upstream of ermC). (TIFF) [file pone.0128017.s002.tiff]

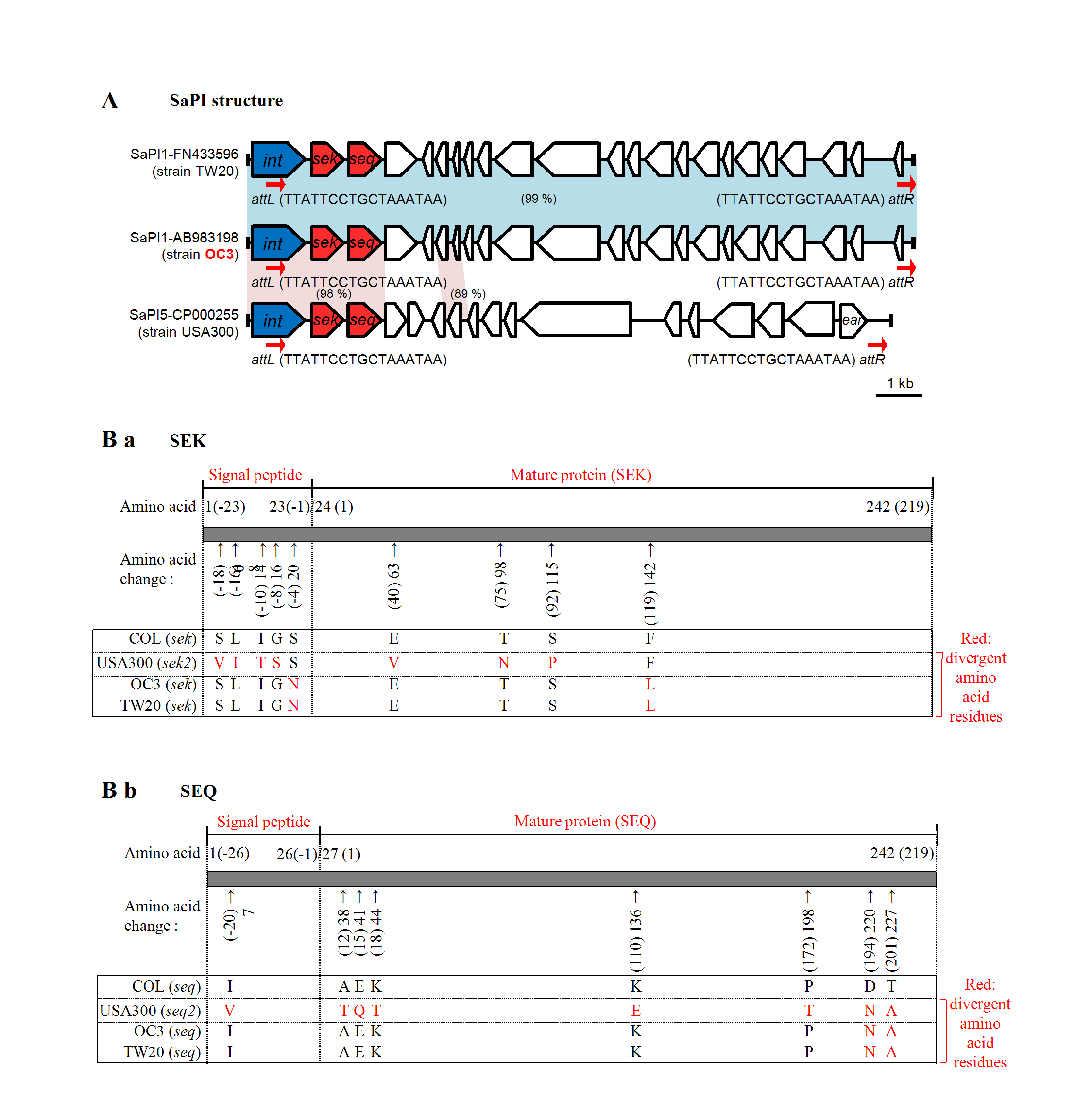

Supplement: S3 Fig — In A, SaPI1 (OC3) showed the highest homology to SaPI1 (TW20). SaPI1 (OC3) was also compared with SaPI5 (USA300). Homologous regions between the SaPI structures are shaded with color. ear, penicillin-binding protein fragment. In B, the deduced amino acid sequences of the sek and seq genes (of OC3, TW20, and USA300) were compared with those of COL. Arrows indicate the positions of the amino acid changes. Different amino acids from the amino acid sequences of COL are indicated in red letters. (TIFF) [file pone.0128017.s003.tiff]

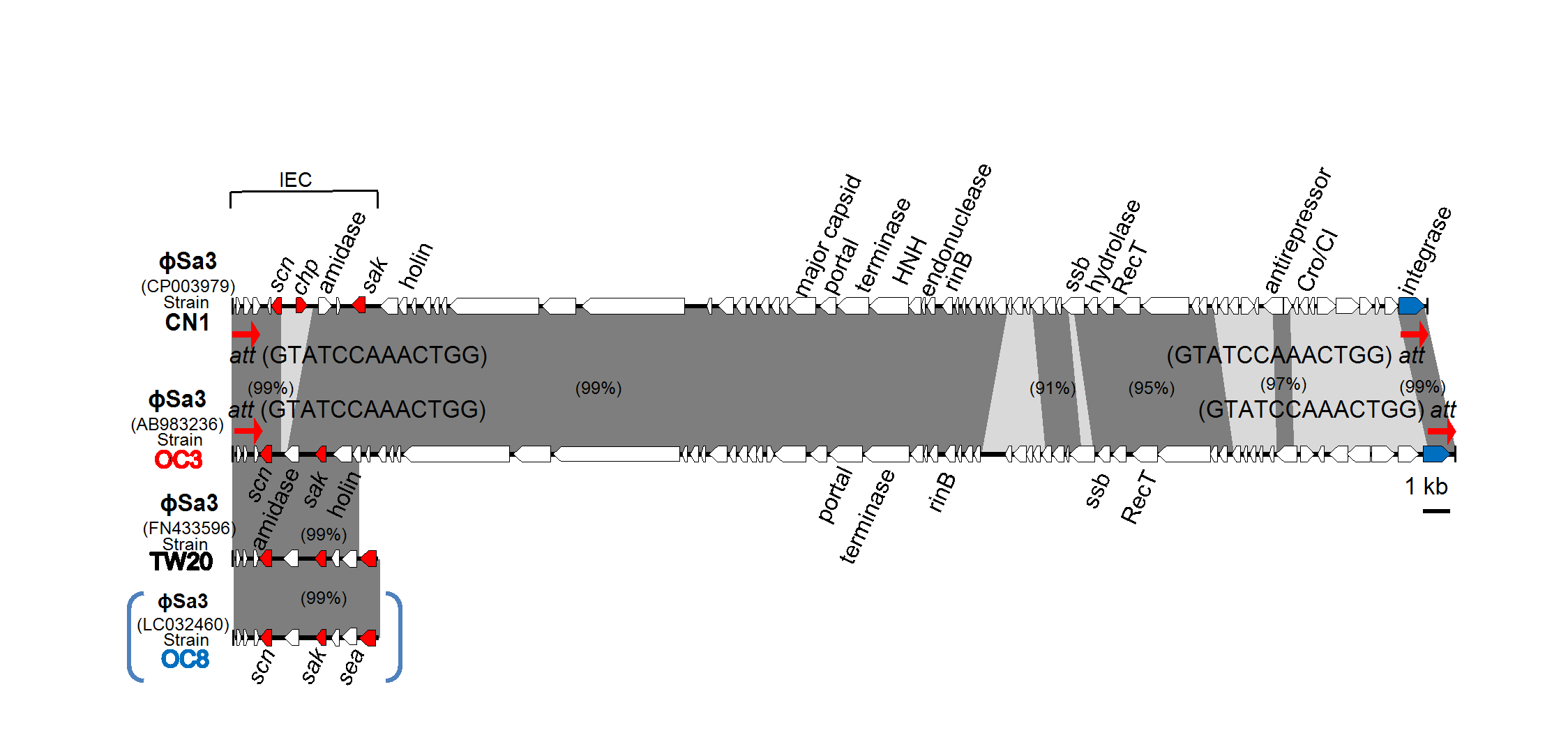

Supplement: S4 Fig — φSa3 (OC3) exhibited the highest homology to φSa3 (CN1). The left-side immune evasion cluster (IEC) region was also compared with those of φSa3 (TW20) and ST8Kras strain OC8. Homologous regions are shaded in each comparison. Genes in IEC: scn, staphylococcal complement inhibitor (SCIN) gene; chp, chemotaxis inhibitory protein of S. aureus (CHIPS) gene; sak, staphylokinase (SAK) gene; sea, staphylococcal enterotoxin A (ETA) gene. The IEC region, carrying scn and sak, of OC3 (a region from attL to sak) was 3,541 bp in size, and showed 99% homology to the corresponding region of TW20. The IEC region, carrying scn, sak, and sea, of OC8 (a region from attL to sea) was 6,022 bp in size, and showed 99.3% homology to the corresponding region of TW20. (TIFF) [file pone.0128017.s004.tiff]

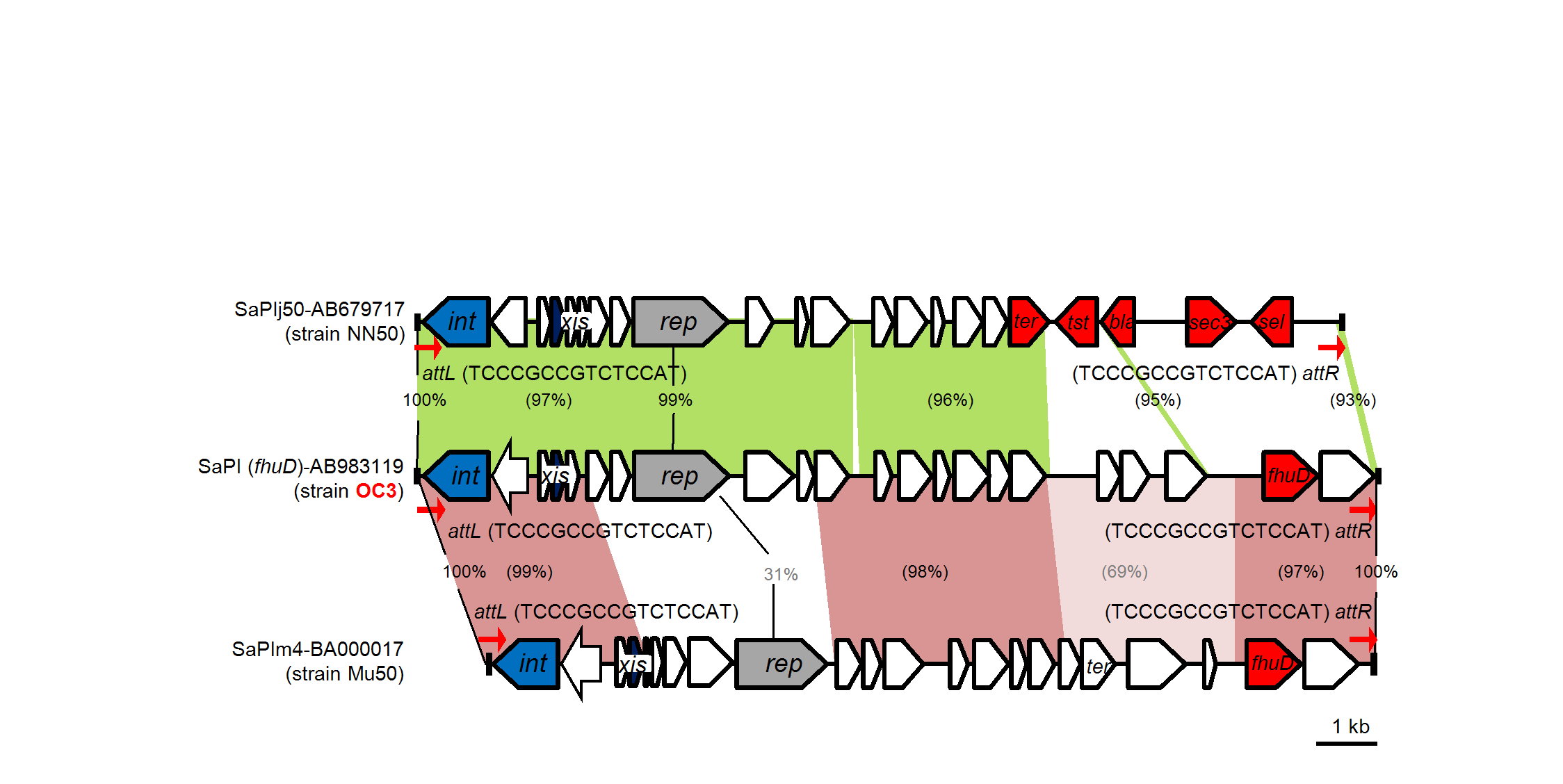

Supplement: S5 Fig — fhuD + SaPI (OC3) showed only limited homology to any previous SaPI, suggesting a novel mosaic SaPI (fhuD). Homologous regions are shaded in each comparison. Genes: int, integrase gene; xis, excisionase; rep, replication initiator gene; ter, terminase gene (which cleaves multimeric DNA); fhuD, ferrichrome ABC transporter homologue. (TIFF) [file pone.0128017.s005.tiff]

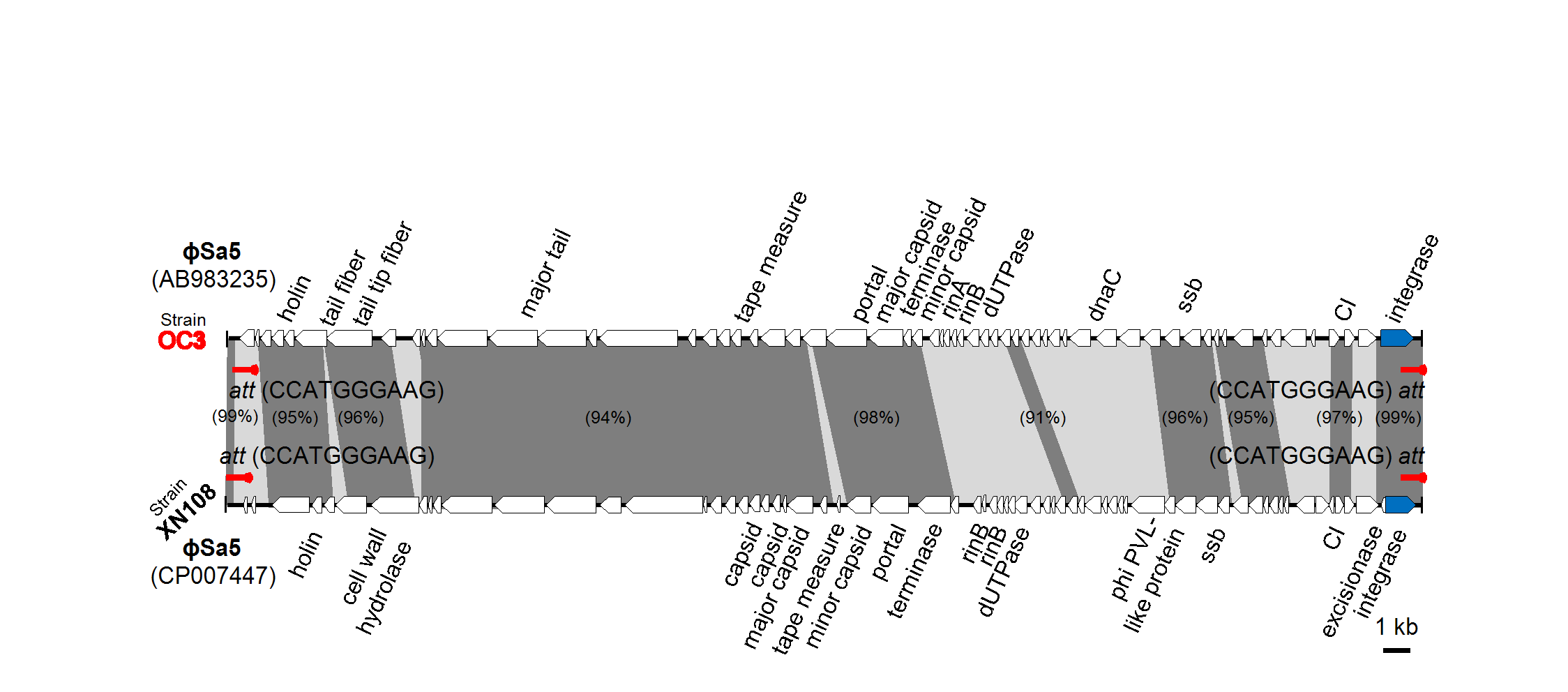

Supplement: S6 Fig — φSa5 (OC3) exhibited the highest (but limited) homology to φSa5 (XN108), suggesting a new mosaic phage. Homologous regions are shaded in each comparison. (TIFF) [file pone.0128017.s006.tiff]

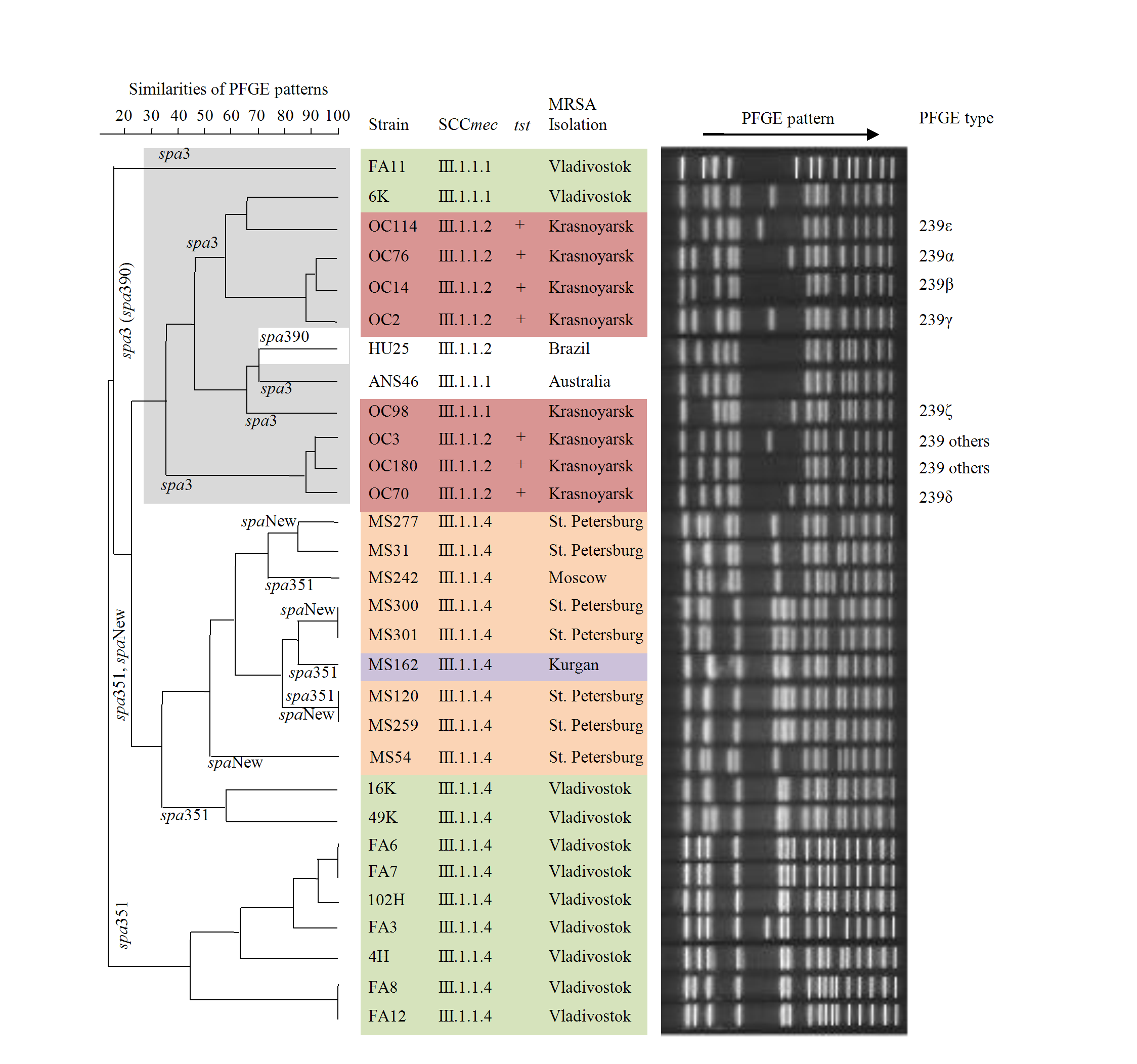

Supplement: S7 Fig — In the dendrogram (left side), a large spa3/SCCmecIII.1.1.1-III.1.1.2 cluster, associated with the Siberian region and Far Eastern region, is shadowed. In the middle of the figure, each Russian region is distinguished by color: red, Siberian region (Krasnoyarsk); green, Far Eastern region (Vladivostok); brown, European region (Moscow, St. Petersburg), purple, Ural region (Kurgan). Reference strains (HU25 and ANS46) are not marked. Regarding PFGE patterns (right side), the PFGE types of ST239 MRSA from Krasnoyarsk are those shown in Fig 1. (TIFF) [file pone.0128017.s007.tiff]

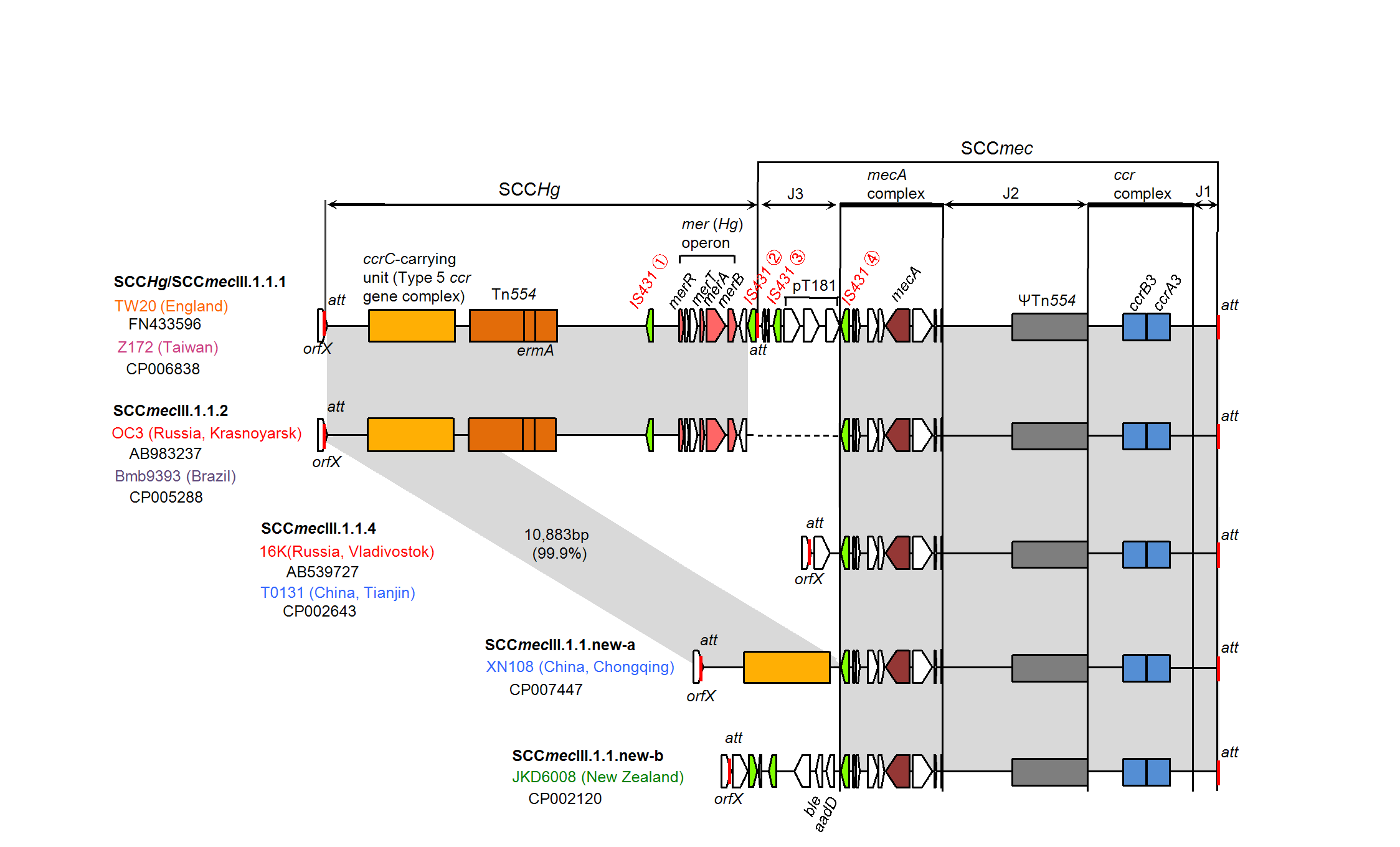

Supplement: S8 Fig — Eight whole genome-analyzed ST239 strains, shown in this figure, are those described in S2 Table. SCCmecIII structures were analyzed as shown in Fig 3. Homologous regions are shaded. SCCmecIII.1.1.new-a and SCCmecIII.1.1.new-b, SCCmecIII.1.1 with new J3 regions. (TIFF) [file pone.0128017.s008.tiff]

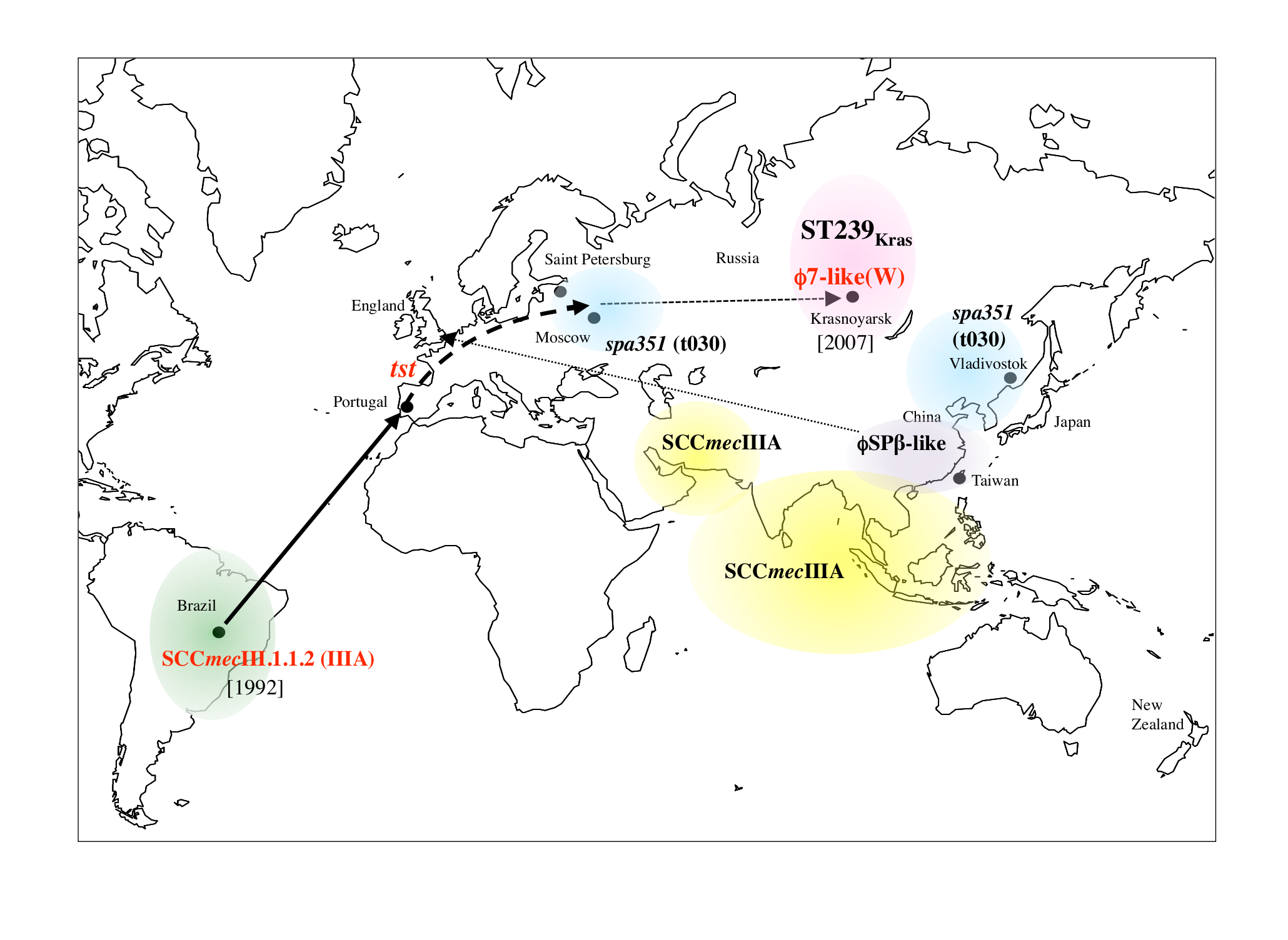

Supplement: S9 Fig — ST239Kras is characterized by SCCmecIII.1.1.2, tst, and φSa7-like (W). This figure indicates a possible Brazil-Europe-Russia transmission route for ST239Kras, in addition to the territories of some other prevalent ST239 MRSA. (TIFF) [file pone.0128017.s009.tiff]
